# Targeting arginine metabolism overcomes chemotherapy resistance in aggressive-variant prostate cancers

**DOI:** 10.1016/j.isci.2026.116184

**Published:** 2026-06-02

**Authors:** Elavarasan Subramani, Patrick G. Pilié, Rebecca Slack-Tidwell, Paul V. Viscuse, Xianghong Kuang, Thirukumaran Kandasamy, Dominik Awad, Jenny J. Han, Licai Huang, Christine B. Peterson, Amado J. Zurita, Sumit K. Subudhi, Paul G. Corn, Rama Soundararajan, Peter Shepherd, Badrajee Piyarathna, Vasanta Putluri, Nagireddy Putluri, Arun Sreekumar, Yuzhuo Wang, Amina Zoubeidi, Iqbal Mahmud, Sara A. Martinez, Lin Tan, Philip L. Lorenzi, Sreyashi Basu, Sonali Jindal, Padmanee Sharma, Christopher J. Logothetis, Timothy C. Thompson, Daniel E. Frigo, Ana M. Aparicio

**Affiliations:** 1 Department of Cancer Systems Imaging, The University of Texas MD Anderson Cancer Center, Houston, TX, USA; 2 Department of Genitourinary Medical Oncology, The University of Texas MD Anderson Cancer Center, Houston, TX, USA; 3 Department of Biostatistics, The University of Texas MD Anderson Cancer Center, Houston, TX, USA; 4 Department of Medicine, University of Virginia, Charlottesville, VA, USA; 5 Department of Statistics, Rice University, Houston, TX, USA; 6 Department of Translational Molecular Pathology, The University of Texas MD Anderson Cancer Center, Houston, TX, USA; 7 Department of Molecular and Cellular Biology, Baylor College of Medicine, Houston, TX, USA; 8 Dan L Duncan Comprehensive Cancer Center and Advanced Technology Cores, Baylor College of Medicine, Houston, TX, USA; 9 Department of Urologic Sciences, University of British Columbia, Vancouver, BC, Canada; 10 Metabolomics Core Facility, Department of Bioinformatics and Computational Biology, The University of Texas MD Anderson Cancer Center, Houston, TX, USA; 11 Department of Hematology and Hematopoietic Cell Transplantation, City of Hope National Medical Center, Duarte, CA, USA; 12 Immunotherapy Platform, James P. Allison Institute, The University of Texas MD Anderson Cancer Center, Houston, TX, USA; 13 Department of Immunology, The University of Texas MD Anderson Cancer Center, Houston, TX, USA; 14 Center for Nuclear Receptors and Cell Signaling, University of Houston, Houston, TX, USA; 15 Department of Biology and Biochemistry, University of Houston, Houston, TX, USA

**Keywords:** Health sciences, Medicine, Medical specialty, Oncology

## Abstract

Aggressive-variant prostate cancers (AVPCs) respond poorly to anti-androgen therapy but show sensitivity to taxane-platinum chemotherapy, though outcomes remain poor. We conducted a phase 2 trial testing induction cabazitaxel plus carboplatin (CabCarb) followed by olaparib maintenance versus observation in men with AVPC. The primary endpoint of improved progression-free survival (PFS) was not met, likely due to the study being underpowered after 38.5% of patients experienced early progression (ChemoPD) prior to randomization. No genomic alterations predicted ChemoPD; however, transcriptomic analysis revealed the enrichment of metabolic pathways, including arginine metabolism, in ChemoPD tumors. These findings were supported by metabolomics data from preclinical models. In AVPC models, arginine depletion with ADI-PEG20 enhanced CabCarb efficacy *in vitro* and *in vivo*. Together, these results provide insight into the heterogeneity of AVPCs and establish a rationale for novel combination treatment strategies to overcome chemotherapy resistance.

## Highlights

• Platinum chemotherapy response in AVPCs is heterogeneous
• Altered arginine metabolism is a hallmark of aggressive-variant prostate cancers
• Arginine depletion using ADI-PEG20 sensitizes AVPCs to platinum-based chemotherapy
• ADI-PEG20 causes compensatory arginine biosynthesis that platinum can impair

## Graphical abstract

**Figure:**
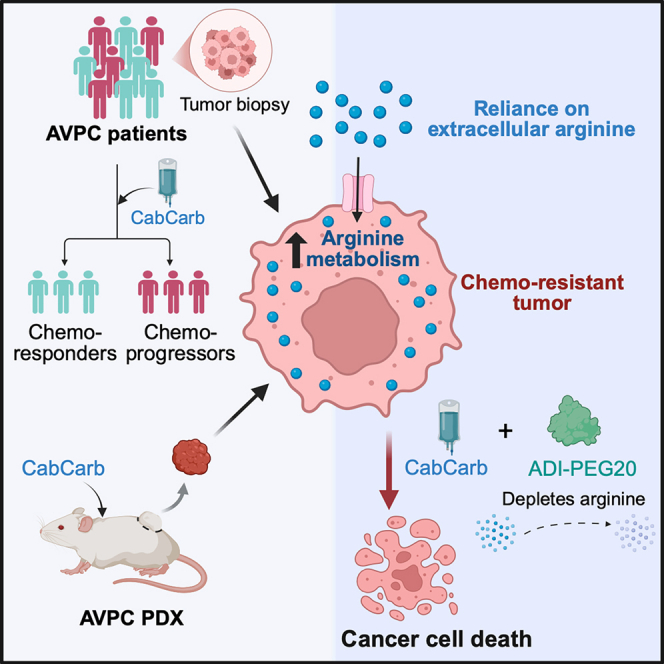

## Introduction

Despite the notable heterogeneity in outcomes for patients with advanced prostate cancers, which largely hinges on response to androgen signaling inhibition,^1^^,^^2^ current treatment algorithms continue, for the most part, to reflect a one-size-fits-all approach. This approach results in dismal outcomes for lethal prostate cancers that respond poorly to androgen signaling inhibition, aka the “androgen-indifferent” prostate cancers, the biology of which remains poorly understood. The lack of reliable biomarkers that can identify “androgen-indifferent” prostate cancers is a major obstacle toward developing therapies specific to these tumors’ biology. To address this limitation and enrich for androgen-indifferent disease in prospective clinical trials, we compiled the aggressive-variant prostate cancer (AVPC) criteria,^3^^,^^4^ a set of clinicopathological and molecular features frequently associated with the rare, androgen-resistant, small-cell, or poorly differentiated high-grade neuroendocrine prostate cancer (NEPC) histological variants.^3^^,^^5^ These criteria include the presence of exclusive visceral metastases, predominantly lytic bone metastases, bulky tumor masses, high soluble levels of carcinoembryonic antigen (CEA), high lactate dehydrogenase (LDH) production, low serum prostate-specific antigen (PSA) production relative to tumor burden, short time to anti-androgen resistance, and combined defect in the TP53, RB1, and/or PTEN tumor suppressors.^1^^,^^3^^,^^4^^,^^5^^,^^6^^,^^7^ In phase 2 clinical trials, we showed that men with prostate cancers meeting at least one of the AVPC clinical criteria benefited from the addition of carboplatin to taxane chemotherapy.^3^^,^^8^ However, responses remained short and overall outcomes poor, which highlighted the need to develop new therapeutic strategies to prolong progression-free survival (PFS) and overall survival (OS) in men with AVPCs.

We had shown in preclinical models that AVPC tumors have aberrant DNA damage repair (DDR) pathway activation,^9^ which has been linked to responsiveness to the PARP inhibitor (PARPi) olaparib.^10^ Indeed, PARPi have shown benefit in the highly replicative, small-cell cancers from various organ sites,^3^^,^^8^^,^^11^^,^^12^^,^^13^ and, as shown in ovarian cancer,^14^^,^^15^ platinum sensitivity itself can serve as a predictor for benefit from PARPi. Thus, we conducted a tissue-rich, randomized trial for men with AVPC (NCT03263650) to test the hypothesis that PARPi (olaparib) maintenance following cabazitaxel plus carboplatin (CabCarb) would improve their PFS. Although a subset of patients did derive long-term benefit from this treatment strategy, over one-third progressed while receiving CabCarb. In this study, we sought to understand the mechanisms underlying this resistance and develop clinically applicable strategies to overcome it.

## Results

### Clinical trial results

**Figure 1:**
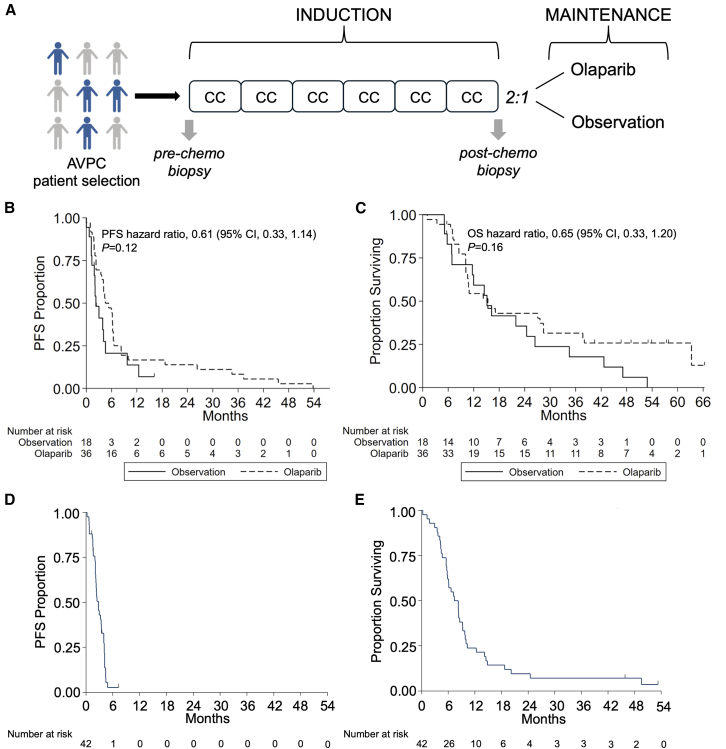
Trial schema and survival outcomes for NCT03263650 (A) Trial schema for NCT03263650. Patients with aggressive-variant prostate cancer (AVPC) were treated with six initial cycles of cabazitaxel + carboplatin (CabCarb) chemotherapy, followed by a two-to-one randomization to olaparib vs. observation maintenance. (B and C) PFS (B) and OS (C) from time of randomization. (D and E) PFS (D) and OS (E) for the 42 patients that were not randomized due to disease progression (*n* = 35) and toxicity or other reasons (*n* = 7).

Between October 2017 and August 2020, 96 patients meeting at least one of the AVPC criteria^1^^,^^3^^,^^4^^,^^8^ (Table S1) started receiving induction chemotherapy with CabCarb (Figure 1A). Baseline characteristics are shown in Table S2. Of the 96 patients, 35 (36.4%) had cancer progression prior to being eligible for randomization, and 7 (7.3%) went off study for reasons other than cancer progression (Figure S1). In addition, 2 patients were found to have progression on CabCarb after further examination of all lines of evidence collected before randomization but interpreted after randomization; so, those patients were included in the ChemoPD group. Thus, 52 of the 54 randomized patients went on to receive randomized treatment with olaparib (*n* = 35) or observation (*n* = 17) after receiving up to 6 cycles (∼4.5 months) of CabCarb (Figure S1). Post-hoc exploratory analyses showed that patients with ChemoPD (*n* = 37) were more likely to have received prior docetaxel and have bone metastases, lower hemoglobin and albumin, and higher LDH and alkaline phosphatase (Alk Phos) compared with those “randomized and treated” (Table S3).

For the patients reaching randomization (with a median follow-up time of 53.9 months post-randomization), the median PFS from randomization was 2.3 (95% confidence interval: 1.3, 4.6) months for observation and 4.9 (3.5, 6.3) months with olaparib for a hazard ratio of 0.61 (0.33, 1.14; *p* = 0.12) (Figure 1B). Median OS from randomization was 15.1 (6.8, 26.4) months for observation and 15.3 (10.2, 28.4) months with olaparib for a hazard ratio of 0.65 (0.33, 1.20; *p* = 0.16) (Figure 1C). PFS (Figure 1D) and OS (Figure 1E) graphs are also shown for the 42 patients who did not reach randomization (induction only), highlighting their virulent disease course. PSA and response evaluation criteria in solid tumors (RECIST) responses are shown in Table S4. Although no unexpected treatment-emergent adverse events (TEAEs) were observed (Table S5), it is noteworthy that during the induction phase, 92 of 96 (96%) treated patients experienced a TEAE, with 44 (46%) having at least one grade ≥3 TEAE. The most common grade ≥3 TEAEs were anemia (*n* = 15, 16%) and thrombocytopenia (*n* = 8, 8%), but 2 (2%) patients suffered grade 4 sepsis, and 1 (1%) patient died of unspecified causes.

In search of predictors of response or resistance, we retrospectively reviewed pathology reports and results from clinical DNA sequencing and targeted immunohistochemistry (IHC) of metastatic biopsies for study participants (Figure S2). Of 95 patients whose tumor biopsies obtained within 2 years of registration, 55 (57.9%) were described as having adenocarcinomas, 35 (36.8%) as having carcinomas, and 5 (5.3%) as having small-cell or high-grade neuroendocrine cancers (NEPCs). Of those with NEPCs, only one reached randomization. Sixty-three (65.6%) patients had clinical germline and/or somatic next-generation sequencing (NGS), and 20 (20.8%) had IHC results for TP53, RB1, and/or PTEN (Table S6). Although numbers are small, we did not observe any substantial associations between the AVPC molecular profile (i.e., combined defects in TP53, RB1, and/or PTEN status) or alterations in DDR genes with ChemoPD or PFS. However, there was a modest (*p* = 0.03) inverse correlation between the AVPC IHC^+^ signature and alterations in DDR genes, further suggesting that other processes might have been driving chemotherapy resistance in this population (Table S6).

### Enrichment of dysregulated arginine metabolism in therapy-resistant AVPCs

**Figure 2:**
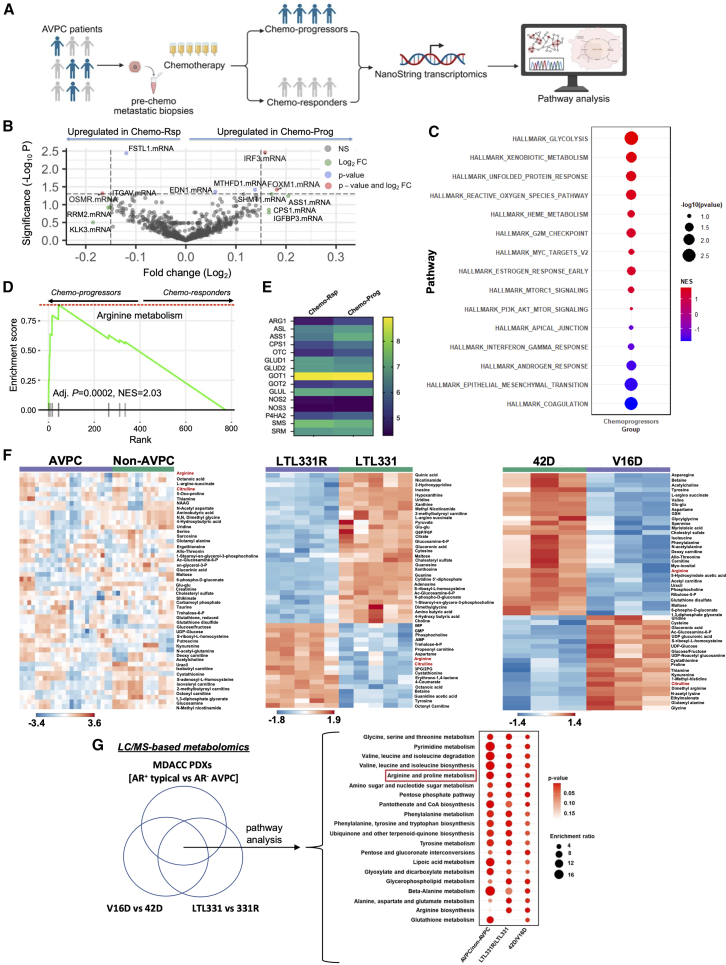
Transcriptome analysis of metastatic tumor biopsies and metabolomics analyses of preclinical models highlight altered arginine metabolism as a hallmark of therapy-resistant AVPCs (A) Pre-chemotherapy metastatic biopsies were subjected to NanoString transcriptome analysis. (B) Volcano plot of NanoString analyses comparing transcriptomic data of chemo-progressors (Chemo-Prog, *n* = 26) versus prolonged chemo-responders (>12 months; Chemo-Rsp, *n* = 25). (C) HALLMARK GSEA of Chemo-Prog versus prolonged Chemo-Rsp transcriptomic data. Only significantly (nominal *p* < 0.05) altered HALLMARK pathways are shown. (D) Enrichment in altered arginine metabolism transcriptomic signature in the Chemo-prog versus Chemo-Rsp group. (E) Heatmap of mean expression values for all arginine signature genes from NanoString analysis. (F) Heatmap of top 50 altered metabolites from unbiased comparison of LC-MS metabolomic profiles in (i) previously characterized MDACC PDXs (*n* = 4/PDX) of adenocarcinoma, non-AVPC (MDA-PCa-133-4, -170-1, 183, 274), and AVPC (MDA-PCa-144-13, 155-2, 177-0, 205-6, 166-1, and 146-10) models (Soundararajan et al.^16^; *n* = 3/PDX); (ii) treatment-induced isogenic LT331 to LTL331R PDX series (Lin et al.^17^; *n* = 5/PDX); and (iii) treatment-induced isogenic V16D to 42D cell models (Bishop et al.^18^; *n* = 3/cell line). (G) Pathway analysis of metabolomics described in (F).

Of the 91 samples with gene expression profiling results, 39 (42.9%) belonged to patients in the ChemoPD group (26 obtained before chemotherapy and 13 post-chemotherapy) and 52 (57.1%) to patients who were randomized (25 obtained before chemotherapy and 27 post-chemotherapy). Given the lack of obvious differences in baseline genomic aberrations or IHC markers in ChemoPD vs. randomized patients, we next explored the transcriptomic differences in available pre-CabCarb metastatic tumor biopsies from patients with ChemoPD (*n* = 26) versus those randomized (chemo-responders; *n* = 25) to further look for predictors of chemotherapy response (Figures 2A and 2B). Possibly due to the limited sample size, low RNA, and/or the heterogeneity of AVPC, relatively few genes were significantly differentially expressed between the two groups on an individual gene basis (Figure 2B). Hence, we next looked for subtle pathway changes using gene set enrichment analysis (GSEA) of the HALLMARK pathways, which revealed upregulation of metabolic pathways in the ChemoPD group (Figure 2C; Table S7). Within the HALLMARK_XENOBIOTIC_METABOLISM, we found multiple leading-edge genes linked to arginine metabolism (Table S7). When we analyzed the data using KEGG pathway signatures, we found aberrant arginine metabolism to be the most dysregulated pathway (Figures 2D and 2E). This finding is aligned with those of prior work in other tumor types, including small-cell lung cancer, where arginine metabolism, which plays critical roles in the regulation of DNA replication, DNA repair, and cell proliferation,^19^ has been implicated in resistance to platinum-based chemotherapy.^20^^,^^21^^,^^22^^,^^23^^,^^24^

We observed convergent mechanisms of therapy resistance in parallel preclinical studies. First, we performed liquid chromatography-mass spectrometry (LC-MS)-based unbiased metabolomics on three different sets of preclinical models that have been well characterized to exhibit treatment-refractory AVPC or non-AVPC (classic adeno-mCRPC) phenotypes^25^^,^^26^^,^^27^^,^^28^: (1) characterized AVPC versus adeno-mCRPC patient-derived xenograft (PDX) models; (2) a treatment-induced isogeneic model of adeno-CRPCàNEPC/AVPC transition; and (3) an isogenic cell model of adeno-CRPCàNEPC/AVPC transition developed from treatment-induced adeno-CRPC and matched NEPC/AVPC xenografts (Figure 2F). The latter two isogenic models served as orthogonal systems that increased confidence that the differences observed in the AVPC versus adeno-mCRPC PDX comparison were biologically meaningful, while also helping to account for the disease heterogeneity inherent to the relatively small PDX cohorts (*n* = 4–6 models per group). Pathway analysis also revealed that arginine metabolism was among the most significantly altered pathways (upregulated and downregulated metabolites) consistently in all three AVPC versus non-AVPC cohort model comparisons (Figure 2G; Table S8).

**Figure 3:**
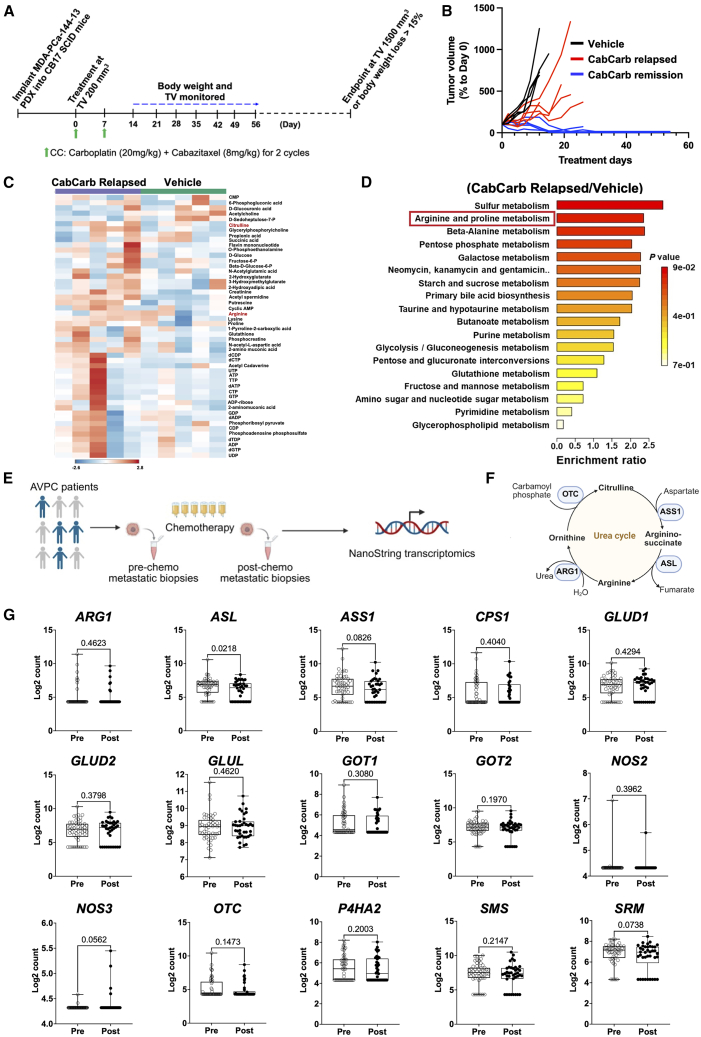
Arginine metabolism is dysregulated in chemotherapy-resistant AVPC preclinical models, and platinum-based chemotherapy decreases expression of *de novo* arginine metabolism genes in AVPC patient tumors (A) Treatment schema of mice bearing MDA-PCa-144-13 subcutaneous PDXs and treated with vehicle (*n* = 5) or cabazitaxel (8 mg/kg) + carboplatin (20 mg/kg) (CabCarb; *n* = 10). TV = tumor volume. (B) Individual tumor growth curves of MDA-PCa-144-13 PDXs. Drug-treated mice were classified into relapsed (red) or remission (blue) groups (*n* = 5) based on response to CabCarb therapy. Data are expressed as % change from the baseline tumor volume. (C) IC-MS metabolomics were performed on fresh frozen samples isolated from vehicle or CabCarb-treated and relapsed tumors described in (B). Top 50 metabolites are shown in the heatmap. Expanded metabolomics data are presented in Table S8. (D) Unbiased enrichment analysis of metabolic alterations in relapsed tumors relative to vehicle-treated tumors. (E) Pre- chemotherapy (*n* = 51) and post-chemotherapy (*n* = 38) metastatic biopsies were subjected to NanoString transcriptome analysis. (F) Schematic of arginine biosynthesis. Major enzymatic reactions are highlighted. Abbreviations: ARG1, arginase-1; ASL, argininosuccinate lyase; ASS1, argininosuccinate synthetase 1; OTC, ornithine transcarbamylase. (G) NanoString analyses of arginine pathway mRNAs from pre- and post-chemotherapy-treated tumor biopsies. Normalized mRNA values of 4.3219281 are below the limit of detection. Welch’s *t* test *p* values are shown. Boxes represent the first and third interquartile ranges.

Next, we asked whether altered metabolism correlated with CabCarb treatment response in mice harboring MDA-PCa-144-13 PDX tumors (Figure 3A). MDA-PCa-144-13, an AR-negative NEPC, is one of eight sublines (four with NEPC morphology, and four with large-cell neuroendocrine carcinoma morphology) derived from the salvage pelvic exenteration specimen of a patient previously treated on a single-arm phase 2 clinical trial of carboplatin and docetaxel, followed by salvage cisplatin and etoposide upon progression.^3^^,^^29^ We observed marked heterogeneity in the PDX’s response to therapy and tumor relapse post CabCarb treatment (Figure 3B), akin to what we observed in the clinical trial participants. Metabolomics analysis of the PDX tumors revealed significant upregulation of arginine metabolism in the CabCarb-relapsed group compared with the vehicle control, among other metabolic pathways (Figures 3C and 3D; Table S9), highlighting a potential targetable pathway in combination with chemotherapy in this AVPC subgroup.

### Arginine depletion sensitizes AVPC models to platinum-based chemotherapy

Arginine metabolism is an emerging, targetable dysregulated pathway implicated in sensitivity and resistance to DNA-damaging platinum agents in other tumor types.^23^^,^^24^^,^^30^^,^^31^ Previous preclinical and clinical studies in cancer have shown the benefit of arginine depletion/starvation when it is given along with platinum chemotherapy.^21^^,^^22^ This effect is thought to be mediated, at least in part, by the downregulation of argininosuccinate synthetase 1 (ASS1) and possibly additional urea cycle-related enzymes in platinum-treated tumors, which results in arginine auxotrophy (reliance on exogenous sources of arginine). In support of this hypothesis and despite the above-noted limitations in sampling, we observed a trend toward lower median levels of *ASS1* and *ASL* expression in post-CabCarb patient samples relative to those in matched pre-CabCarb samples, suggesting an increased reliance on exogenous sources of arginine because of chemotherapy treatment (Figures 3E–3G).

**Figure 4:**
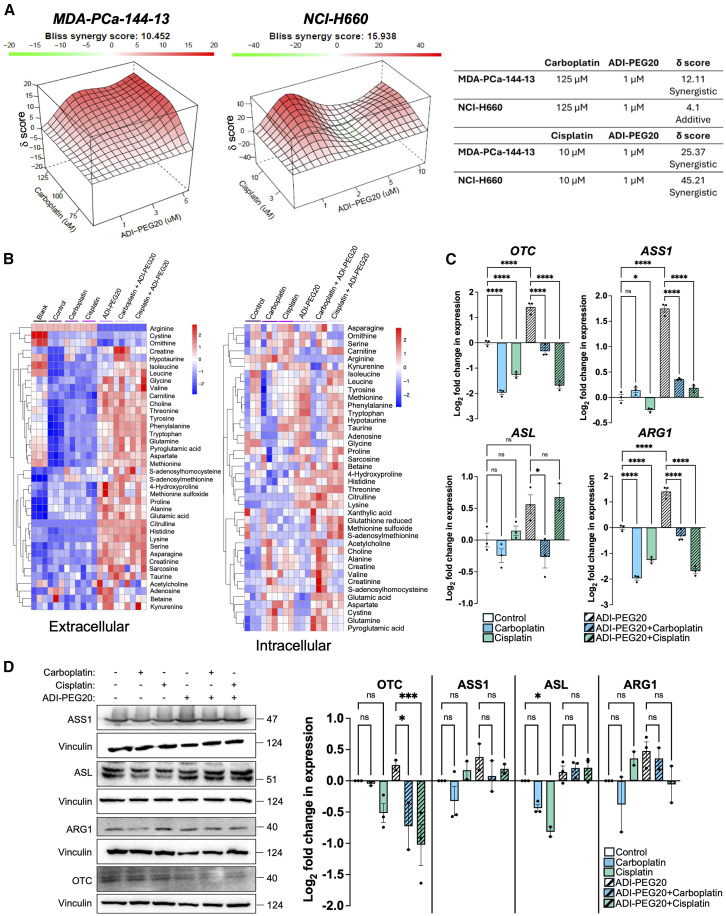
Combination treatment of ADI-PEG20 with platinum chemotherapy induces synergistic anti-cancer effects in preclinical cell models of AVPC (A) AVPC MDA-PCa-144-13 or NCI-H660 cell models were treated for 3 days with vehicle or increasing concentrations of carboplatin or cisplatin ± increasing doses of ADI-PEG20. The cells were then subjected to resazurin-based cell survival assays (*n* = 3). Bliss synergy calculations were based on percent killing. Bliss scores (δ score) > 10 indicate synergistic cell killing. Representative Bliss synergy plots (left) and single-point synergy scores (right) are shown. Complete dose responses are shown in Figure S3. (B) MDA-PCa-144-13 cells were treated with carboplatin (125 μM), cisplatin (10 μM), and/or ADI-PEG20 (1 μM) for 72 h and subjected to IC-MS metabolomics (*n* = 3/group). Amino acid profiles are shown here. Expanded metabolomics data are shown in Figure S4 and Table S10. (C and D) MDA-PCa-144-13 cells were treated, as in (B), for 24 h (C) or 48 h (D), following which RNA (C) or protein lysates (D) were collected and subjected to RT-qPCR (C) or immunoblot (D; left, representative images; right, densitometry) (*n* = 3). Data are presented as the mean ± SE and were analyzed using ANOVA and Tukey’s post-hoc test. ∗*p* < 0.05; ∗∗∗*p* < 0.001; ∗∗∗∗*p* < 0.0001; ns, not significant.

To functionally test whether arginine depletion could improve platinum-based chemotherapy in AVPC, we treated the AR-negative NEPC cell lines NCI-H660 and MDA-PCa-144-13, both of which exhibit small cell morphology and harbor RB1 deficiency and TP53 mutations,^28^^,^^29^^,^^32^ with platinum chemotherapy with or without increasing doses of the pegylated arginine deiminase ADI-PEG20 (pegargiminase) and observed synergistic cell killing from this combination treatment (platinum chemotherapy + ADI-PEG20) in both NCI-H660 and PDX 144-13 cells (Figures 4A and S3). To further define the metabolic consequences of platinum-based chemotherapy in AVPC, we performed metabolomic, mRNA, and protein expression analyses in MDA-PCa-144-13 cells. Metabolomic profiling revealed a marked reduction in the arginine levels in both culture media (extracellular) and intracellular compartments following ADI-PEG20 treatment, accompanied by a corresponding increase in citrulline, which confirmed enzymatic conversion of arginine to citrulline (Figure 4B; Table S10). Notably, ornithine levels were reduced in the cultured media relative to media-only controls, indicating active uptake of ornithine by AVPC cells (Figure 4B). Consistent with this observation, the expression of key urea cycle enzymes involved in arginine biosynthesis, including ASS1, ASL, and OTC was found to be increased following ADI-PEG20 treatment, suggesting the engagement of compensatory *de novo* arginine biosynthesis (Figures 4C and 4D). In contrast, combining ADI-PEG20 with carboplatin or cisplatin largely attenuated this compensatory induction, leading to reduced ornithine availability, decreased fumarate, and accumulation of intracellular aspartate (consistent with impaired ASS1 activity), along with suppressed expression of central arginine biosynthesis enzymes (Figures 4B–4D and S4). Together, these effects indicate impaired arginine restoration under platinum-induced genotoxic stress.

**Figure 5:**
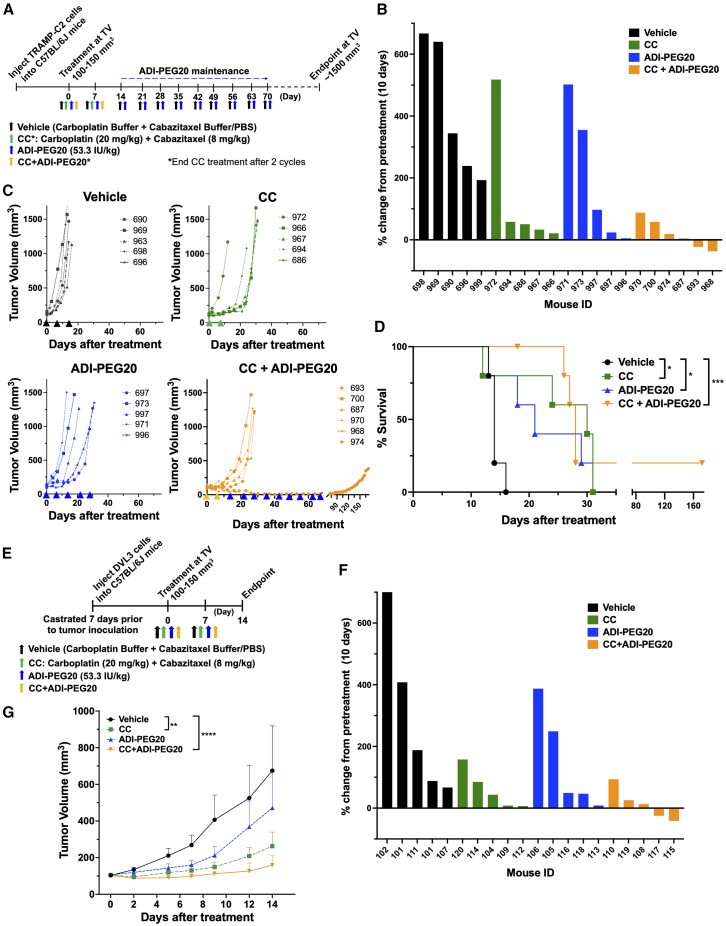
ADI-PEG20 sensitizes preclinical models of AVPC to platinum chemotherapy *in vivo* (A) Treatment schema of the mice bearing TRAMP-C2 tumors and treated with vehicle (*n* = 5), cabazitaxel + carboplatin (CabCarb, *n* = 5), ADI-PEG20 (*n* = 5), and CabCarb + ADI-PEG20 (*n* = 6). (B) Waterfall plot of % change in TRAMP-C2 tumor volume following treatment. (C) Tumor growth curves of individual mice over time following treatment. Numbers in legends denote mouse IDs. Arrows on *x* axis refer to the treatments described in (A). (D) Survival curves comparing different treatment groups. The mice were sacrificed when tumors reached >1,500 mm^3^. Log-rank test; ∗*p* < 0.05, ∗∗∗*p* < 0.001. (E) Treatment schema of castrated mice bearing DVL3 tumors and treated with vehicle (*n* = 5), CC (*n* = 5), ADI-PEG20 (*n* = 5), or CC + ADI-PEG20 (*n* = 5). (F) Waterfall plot of % change in DVL3 tumor volume following treatment. (G) DVL3 tumor growth curves of mice over time following treatment. Data are presented as mean tumor volume ± SEM. Two-way ANOVA and Dunnett’s test; ∗∗*p* < 0.01, ∗∗∗∗*p* < 0.0001.

Given potential differences between metabolism *in vitro* and *in vivo,*^33^ we next tested the efficacy of ADI-PEG20 alone or in combination with CabCarb in a TRAMP-C2 syngeneic model of advanced prostate cancer (Figures 5A–5D). TRAMP-C2 murine prostate cancer cells were derived from the TRAMP genetically engineered mouse model, which is driven, in large part, by the inactivation of p53 and RB1 and is known to develop NEPC.^34^ As observed in our cell culture-based experiments, ADI-PEG20 improved the response to CabCarb, resulting in impaired tumor growth and prolonged survival (Figures 5B–5D). Likewise, ADI-PEG20 improved the response to CabCarb in a second syngeneic model, DVL3, which harbored dual genetic loss of *Pten* and *Trp53*^35^ (Figures 5E–5G). Together, these data and others^21^^,^^22^^,^^23^^,^^24^^,^^30^^,^^31^^,^^36^^,^^37^ provided the scientific rationale for our novel clinical trial entitled “Phase 1/2 study of PEGylated arginine deiminase (ADI-PEG20) with carboplatin and cabazitaxel in men with AVPC” that is now recruiting (NCT06085729).

## Discussion

In phase 2 clinical trial (NCT03263650) presented here, we did not observe a statistically significant PFS improvement with PARPi maintenance therapy following CabCarb chemotherapy in men with lethal, advanced AVPC (Figure 1), possibly due to the small sample size. However, tissue sampling allowed for exploratory analyses to investigate baseline altered pathways that drive chemotherapy resistance. Interestingly, the heterogeneity in response or resistance to chemotherapy is not clearly explained by pathogenic mutations in cancer-related genes alone (e.g., alterations in *AR*, canonical tumor suppressors, or various DNA repair genes) but hinges largely on differences in the cellular resources and metabolism. Altered metabolic pathways have been linked to chemotherapy resistance and are potentially targetable.^23^^,^^31^^,^^38^ Here, we describe altered arginine metabolism as a hallmark of chemotherapy-resistant AVPC in patients (Figure 2). Furthermore, matched pre- and post-CabCarb tissue sampling in this trial suggests that chemotherapy suppresses *de novo* arginine biosynthesis (Figure 3), which has been previously shown to cause arginine auxotrophy and subsequent sensitivity to extracellular arginine deprivation.^21^^,^^22^^,^^23^^,^^24^^,^^30^^,^^31^^,^^36^^,^^37^ Using preclinical models, we confirmed that AVPCs exhibit altered arginine metabolism, among other metabolic pathways, and that arginine metabolism is implicated in chemo-resistance, as has been shown in other tumor types (Figures 2 and 3).^20^^,^^23^^,^^31^ Because arginine depletion has been shown to overcome resistance to anti-cancer DNA-damaging therapies in selected tumor types and may result in improved responses when combined with platinum-based chemotherapy, we tested whether arginine deprivation using ADI-PEG20 can improve the efficacy of platinum-based chemotherapy in pre-clinical models of AVPC *in vitro* and *in vivo* (Figures 4 and 5). Treatment of human AVPC cell lines and syngeneic mouse models of AVPC with ADI-PEG20 sensitizes AVPCs to CabCarb chemotherapy (Figures 4 and 5). Extending this observation, our integrated metabolomic and mRNA and protein expression analyses in ASS1-intact AVPC models revealed that arginine metabolism is dynamically regulated in response to therapy. Specifically, AVPC cells actively engage urea cycle-mediated arginine regeneration by taking up ornithine and upregulating key biosynthesis enzymes, including ASS1, ASL, and OTC (Figure 4). We speculate that this adaptive response supports survival under platinum-induced genotoxic stress possibly by sustaining arginine-dependent processes required for cellular repair and stress tolerance. Notably, ADI-PEG20 treatment induces a compensatory increase in arginine biosynthesis pathways, whereas combination treatment with platinum chemotherapy suppresses this induction, functionally rendering AVPC cells arginine auxotrophic despite largely retained basal ASS1 expression. Collectively, these findings suggest that platinum chemotherapy disrupts arginine homeostasis not simply by suppressing basal enzyme expression but by interfering with an adaptive metabolic program that cells use in response to cellular and/or metabolic stress. Therapeutic arginine depletion exacerbates this vulnerability by preventing the restoration of intracellular arginine pools, thereby impairing arginine-dependent repair and survival pathways.

At this time, it remains unclear what drives altered arginine metabolism in AVPCs. Although the loss of RB1 and TP53 has been shown to promote AVPC/NEPC,^6^^,^^39^ reanalysis of published RNA sequencing (RNA-seq) data from isogenic LNCaP models in which *RB1*, *TP53*, or both (*RB1* + *TP53*) were knocked out using CRISPR-Cas9 did not reveal any significant changes in the expression of urea cycle enzymes (data not shown).^40^ Notably, this study demonstrated that dual knockout of *TP53* and *RB1* alone was not sufficient to promote NE transdifferentiation, allowing the uncoupling of RB1/TP53 loss from NE signaling. While these data would suggest that neither RB1 nor TP53 directly modulates arginine metabolism, it is important to note that the RNA-seq analyses were conducted using cells grown under nutrient- and arginine-replete conditions. Furthermore, RB1 and/or TP53 may regulate arginine metabolism indirectly through post-transcriptional mechanisms, secondary effects, or interactions with other factors. Additional studies are, therefore, required to determine definitively whether RB1 and/or TP53 regulate arginine metabolism and, if so, under what conditions.

Beyond the upstream drivers of arginine metabolism in AVPC, how exactly this pathway contributes to AVPC biology is still not fully understood. Based on prior studies, arginine’s role in cancer and therapy resistance is likely multifactorial. Within cancer cells, arginine is needed for the production of proteins, polyamines, nitric oxide, and creatine to allow their rapid growth, thereby contributing building blocks, signaling molecules, and energy reserves.^41^^,^^42^ Increased arginine levels also promote mTOR signaling, a known driver of advanced prostate cancer,^43^^,^^44^ through various sensor mechanisms.^45^^,^^46^^,^^47^ While our *in vitro* studies suggest cancer cell-intrinsic roles for arginine in AVPC biology, we cannot rule out cancer cell-extrinsic functions for arginine in the tumor microenvironment. For example, arginine-derived nitric oxide can promote angiogenesis.^48^ Beyond the vasculature, arginine is a major regulator of the immune system. Depletion of arginine by arginase-expressing immunosuppressive cells can impair T cell activity.^49^^,^^50^ In contrast, arginine-mediated nitric oxide production has also been shown to impair the anti-tumor immune response in a context-dependent manner, suggesting that arginine deprivation may sensitize tumors to some immunotherapies.^51^ Interestingly, following ADI-PEG20 treatment, we also observed a slight increase in extracellular adenosine (Figure 4B), which is known to have immunosuppressive effects.^52^^,^^53^ However, platinum chemotherapy decreased extracellular adenosine regardless of the ADI-PEG20 status, suggesting that combination therapies could avoid this potentially immunosuppressive effect (Figure 4B). Whether these additional, non-autonomous mechanisms influence AVPC biology is an area ripe for future investigation. Regardless, in our immune intact, syngeneic models, ADI-PEG20 improved the anti-tumor efficacy of chemotherapy, indicating that systemic arginine deprivation impairs prostate cancer progression. These data, combined with our clinical correlates described above, provide rationale for the design of our ongoing clinical trial of ADI-PEG20 with CabCarb in men with AVPC (NCT06085729).

### Limitations of the study

This study was limited by the small sample size of a known heterogeneous population^54^ and limited scope of our exploratory correlative studies. Nonetheless, through unique trial design incorporating sequential tumor sampling in conjunction with reverse translation and preclinical modeling, we provide data that help unravel the heterogeneity within this patient population with AVPC at the highest risk of dying from prostate cancer as well as rationale for additional treatment combinations to overcome chemotherapy resistance, with plans to validate these findings in subsequent larger patient cohorts treated in studies for predictive biomarker development.

## Resource availability

### Lead contact

Request for further information, resources, and reagents should be directed to and will be fulfilled by the lead contact, Daniel E. Frigo (frigo@mdanderson.org).

### Materials availability

Models generated in this study are available upon request from the corresponding authors.

### Data and code availability

• Complete NanoString data are available in the supplemental information.
• This paper analyzes existing, publicly available RNA-seq data of LNCaP, LNCaP SKO, and LNCaP DKO cell lines, which are available at the Gene Expression Omnibus (GEO), with accession number GEO: GSE147250.
• This paper does not report original code.
• Any additional information required to reanalyze the data reported in this paper is available from the lead contact upon request.

## Acknowledgments

In memoriam of Dr. Nora Navone, we would like to acknowledge her contributions to the work related to PDX model systems in this study. We would like to thank the patients who participated in this clinical trial and their families. We thank Dr. Ian Mills (University of Oxford) for provision of the DVL3 cells. We also thank the MDACC Advanced Technology Genomics Core for NanoString analysis and the MDACC Prostate Cancer PDX Core for PDX models. This work was supported by 10.13039/100000002National Institutes of Health (NIH) grants R01CA283402 (to A.M.A., D.E.F., and P.S.), R01CA281727 (to T.C.T. and P.G.P.), R01CA282282 (to N.P.), the Mike Slive Foundation for Cancer Research (to D.E.F. and A.M.A.), an American Society for Clinical Oncology Conquer Cancer Young Investigator Award (P.V.V.), and the 10.13039/100000892Prostate Cancer Foundation (P.G.P.). This project was also supported by the Cancer Prevention and Research Institute of Texas Proteomics and Metabolomics Core Facility (RP210227), 10.13039/100000002NIH grant P30CA125123, and the 10.13039/100008527Dan L. Duncan Cancer Center.

## Author contributions

Conceptualization, E.S., P.G.P., D.E.F., and A.M.A.; methodology, E.S., P.G.P., R.S.-T., P.V.V., T.K., L.H., C.B.P., C.J.L., T.C.T., D.E.F., and A.M.A.; investigation, E.S., P.G.P., P.V.V., X.K., T.K., D.A., J.J.H., P.S., B.P., V.P., N.P., A.S. I.M., S.A.M., L.T., P.L.L., S.B., S.J., and P.S.; visualization, E.S., P.G.P., R.S.T., P.V.V., X.K., T.K., L.H., C.B.P., R.S., B.P., Y.W., A.Z., I.M., S.B., S.J., D.E.F., and A.M.A.; funding acquisition, P.G.P., P.S., T.C.T., D.E.F., and A.M.A.; project administration, D.E.F. and A.M.A.; supervision, N.P., A.S., P.L.L., P.S., C.J.L., T.C.T., D.E.F., and A.M.A.; writing – original draft, E.S., P.G.P., D.E.F., and A.M.A.; writing – review & editing, E.S., P.G.P., R.S.-T., P.V.V., X.K., T.K., D.A., J.J.H., L.H., C.B.P., A.J.Z., S.K.S., P.G.C., R.S., P.S., B.P., V.P., N.P., A.S., Y.W., A.Z., I.M., S.A.M., L.T., P.L.L., S.B., S.J., P.S., C.J.L., T.C.T., D.E.F., and A.M.A.

## Declaration of interests

P.G.P. has a patent for a replication stress response biomarker. P.V.V. reports consulting for Exelixis, Johnson & Johnson, and Pfizer. A.J.Z. reports consulting with AstraZeneca, Bayer, and Exelixis and has received honoraria from Janssen and Pfizer. AstraZeneca provided funding for the clinical trial NCT03263650. D.E.F. has a familial relationship with March Biosciences, Biocity Biopharmaceuticals, and Barricade Therapeutics. A.M.A. reports consulting for Abbvie, Amgen, Astellas Pharma, AstraZeneca Pharmaceuticals, Bayer, Boehringer-Ingelheim, Bristol-Myers Squibb Pharmaceuticals, Daiichi Sankyo, Genzyme, Janssen Pharmaceuticals, Novartis, Pfizer, and Sanofi, as well as institutional research funding from AstraZeneca Pharmaceuticals, Janssen Pharmaceuticals, and Polaris Pharmaceuticals. Polaris Pharmaceuticals provided the ADI-PEG20 for this study.

## STAR★Methods

### Key resources table

**Table:** 

REAGENT or RESOURCE	SOURCE	IDENTIFIER
**Chemicals, peptides, and recombinant proteins**		

Carboplatin	MedChemExpress	HY-17393
Cabazitaxel	MedChemExpress	HY-15459
ADI-PEG20	Polaris Pharmaceuticals	N/A
Cisplatin	Accord Healthcare Inc.	16729-288-11
Enzalutamide	Selleck Chemicals	S2840
Matrigel® Matrix	Corning	356231
RPMI 1640	Corning	10-040-CV
FBS	Sigma	F4135
Insulin	Gibco	12585014
Apo-Transferrin	Sigma	T5391
Sodium selenite	Sigma	S5261
L-Glutamine	Gibco	25030081
Sodium Pyruvate	Gibco	11360070
HEPES solution	Sigma	H0887
β-estradiol	Sigma	E2257
Hydrocortisone	Sigma	H0135
DMEM	Corning	10-013-CV
Nu-Serum™ IV Growth Medium Supplement	Corning	355504
dihydrotestosterone	Steraloids, Inc	A2570-000
Glucose	Sigma	G8769

**Antibodies**		

Vinculin (E1E9V) Rabbit Monoclonal Antibody	Cell Signaling Technology	13901T; RRID:AB_2728768
ASS1 (D4O4B) XP® Rabbit mAb	Cell Signaling Technology	70720S; RRID:AB_2799790
Arginase-1 (D4E3M™) XP® Rabbit mAb	Cell Signaling Technology	93668S; RRID: AB_2800207
Anti-Ornithine Carbamoyltransferase/OTC antibody, [EPR19725]	Abcam	ab203859; RRID:AB_2876368
Anti-Argininosuccinate Lyase antibody	Abcam	ab97370; RRID:AB_10680261
Anti-rabbit IgG, HRP-linked Antibody	Cell Signaling Technology	7704P2; RRID:AB_2099233

**Oligonucleotides**		

Primer: *ASS1* Forward: GCTTATAACCTGGGATGGGCA	This paper	N/A
Primer: *ASS1* Reverse: CGGAGCCTTTGCTGGACATA	This paper	N/A
Primer: *ASL* Forward: GGCGCGACACTATCCGTG	This paper	N/A
Primer: *ASL* Reverse: CTCCGGGTCTGGAAGACAAG	This paper	N/A
Primer: *ARG1* Forward: TTCACACCAGCTACTGGCAC	This paper	N/A
Primer: *ARG1* Reverse: CCCAGGGATGGGTTCACTTC	This paper	N/A
Primer: *OTC* Forward: CCCAGGACCAACTAGAAAGCC	This paper	N/A
Primer: *OTC* Reverse: CCCCACTACCACATTCTCACA	This paper	N/A
Primer: β-actin Forward: CCACCGCAAATGCTTCTAGG	This paper	N/A
Primer: β-actin Reverse: GTCCTCGGCCACATTGTGAA	This paper	N/A

**Experimental models: Cell lines**		

LNCaP-16D^CRPC^	Dr. Amina Zoubeidi	N/A
LNCaP-42D^ENZR^	Dr. Amina Zoubeidi	N/A
NCI-H660	ATCC	CRL-5813; RRID:CVCL_1576
MDA-PCa-144-13	MDACC Prostate Cancer PDX Core-derived cell line	N/A
DVL3	Dr. Ian Mills	N/A
TRAMP-C2	ATCC	CRL-2731; RRID:CVCL_3615

**Experimental models: Organisms/strains**		

Mouse: CB17/lcr-*Prkdc*^*scid*^/lcrlcoCrl)	Charles River Laboratories	Strain 236; RRID:IMSR_CRL:236
Mouse: C57BL/6J	The Jackson Laboratory	Strain 000664; RRID:IMSR_JAX:000664
LTL331	Dr. Yuzhuo Wang	N/A
LTL331R	Dr. Yuzhuo Wang	N/A
MDA-PCa-133-4	MDACC Prostate Cancer PDX Core	N/A
MDA-PCa-170-1	MDACC Prostate Cancer PDX Core	N/A
MDA-PCa-183	MDACC Prostate Cancer PDX Core	N/A
MDA-PCa-274	MDACC Prostate Cancer PDX Core	N/A
MDA-PCa-144-13	MDACC Prostate Cancer PDX Core	N/A
MDA-PCa-155-2	MDACC Prostate Cancer PDX Core	N/A
MDA-PCa-177-0	MDACC Prostate Cancer PDX Core	N/A
MDA-PCa-205-6	MDACC Prostate Cancer PDX Core	N/A
MDA-PCa-166-1	MDACC Prostate Cancer PDX Core	N/A
MDA-PCa-146-10	MDACC Prostate Cancer PDX Core	N/A

**Critical commercial assays**		

CellTiter-Blue Cell Viability assay	Promega	G808A
Clinical IHC and NGS assays	This paper	N/A
Aurum^TM^ Total RNA Mini Kit	Bio-Rad	7326820
iScript™ cDNA Synthesis Kit,	Bio-Rad	1708890
iQ™ SYBR® Green Supermix	Bio-Rad	1708882
Western Lightning Plus, Chemiluminescent Substrate	Revvity	NEL104001EA
Pierce™ BCA Protein Assay Kits	Thermo Fisher Scientific	23227

**Software and algorithms**		

GraphPad Prism	https://www.graphpad.com/	Version 10; RRID:SCR_002798
nSolver	http://www.nanostring.com/products/nSolver	RRID:SCR_003420
GSEA	https://www.gsea-msigdb.org/gsea/index.jsp	Version 4.3.3; RRID:SCR_003199
R	https://www.r-project.org/	RRID:SCR_001905
ggplot2	https://ggplot2.tidyverse.org/	Version 3.5.2; RRID:SCR_014601
MetaboAnalyst	https://www.metaboanalyst.ca/MetaboAnalyst/	Version 6.0; RRID:SCR_015539
ImageJ	https://imagej.net/ij/	Version 1.54h; RRID:SCR_003070
SynergyFinder	https://synergyfinder.fimm.fi	Version 2.0; RRID:SCR_026127

### Experimental model and study participant details

#### Clinical trial

We conducted an open-label, randomized, single-institution, phase II trial (ClinicalTrials.gov Identifier: NCT03263650) with the primary objective to estimate the progression-free survival (PFS) of men with AVPC treated with 6 cycles of cabazitaxel and carboplatin (CabCarb) followed by olaparib maintenance versus observation. Secondary objectives included: to explore the association between DDR pathway gene expression changes following CabCarb chemotherapy and clinical outcomes; to determine the rate of adverse events attributable to olaparib following CabCarb in men with AVPC; and to estimate the overall survival (OS), RECIST and PSA response rates to CabCarb, and to olaparib maintenance in men with AVPC. The study was conducted in accordance with institutional and federal regulations, including the Declaration of Helsinki, Good Clinical Practice guidelines. The study protocol and its amendments were approved by the MD Anderson Institutional Review Board (IRB). All patients provided written informed consent to participate in this and the M.D. Anderson IRB-approved companion laboratory protocols.

Eligible men had Eastern Cooperative Oncology Group (ECOG) performance status of ≤2, adequate organ function and progressive mCRPC meeting at least one of nine AVPC criteria (see Table S1). To participate in the study, patients had to agree to tissue collection for correlative studies at baseline and at the time of randomization. No prior treatment with carboplatin, cisplatin, cabazitaxel or PARPi was permitted. Patients whose disease was refractory to ≥2 lines of chemotherapy given for CRPC were not eligible. Chronic use of known strong and moderate CYP3A4 inhibitors or inducers was not allowed. Patients with active viral hepatitis or chronic liver disease, a diagnosis or suspicious of myelodysplastic syndrome/acute myeloid leukemia, or history of pneumonitis or extensive bilateral lung disease of non-malignant etiology were also ineligible.

A treatment cycle was defined as a 21-day period. Eligible patients were treated during the induction phase with 6 cycles of intravenous cabazitaxel (25 mg/m^2^), carboplatin (AUC 4) and 5 mg twice daily of oral prednisone on days 1-21 of cycles 1-6. All patients received primary prophylaxis with either filgrastim or pegfilgrastim. If at the treating physician’s discretion, the patient was at high risk for toxicity from additional cycle of chemotherapy, randomization could occur after 4 cycles of chemotherapy. Once chemotherapy was completed, patients were randomized in a 2:1 fashion to treatment with oral olaparib 300 mg twice daily versus observation during the maintenance phase of the study. All drugs could be reduced two times for toxicity (cabazitaxel to 20 mg/m^2^ and 15 mg/m^2^, carboplatin to AUC 3 and AUC 2, prednisone to 5 mg once daily and none, and olaparib to 200 mg and 100 mg twice daily). Restaging studies, including a bone scan and CT or MRI of chest, abdomen and pelvis, were obtained after every 3 cycles of treatment. A resting 12-lead EKG was required prior to start of olaparib or observation, and after every 3 cycles of therapy thereafter.

### Method details

#### Randomization and statistical analysis

A maximum enrollment of 96 patients was planned to have 72 patients randomized to olaparib (n=48) or observation (n=24), assuming that approximately 25% of patients would progress before randomization. There were no stratification factors. The expected median PFS post-randomization in patients assigned to control was 3.9 months based on a landmark analysis of 4.5 months to account for the expected induction time for this study, using preliminary data from a similar patient population.^8^ The choice of 72 randomized patients was designed to detect a difference in median progression-free survival of 7.8 vs 3.9 months for patients receiving olaparib vs control, respectively (hazard ratio 0.50). This assumed a 1-sided 5% significance level and 80% power. This included 2 early looks for futility at 22 and 43 PFS events (at approximately 20 and 32 months after first randomization, respectively), using Lan-DeMets family O’Brien-Fleming type stopping boundaries (East v6). The final analysis was planned once 65 PFS events (expected at approximately 46 months) occurred. This design assumed an accrual rate of 2-3 patients per month, an enrollment period of approximately 33 months and a 12-month period of treatment and follow-up after last subject enrolled. To have sufficient biopsies for the second primary objective, at least 20 patients would need to obtain a second biopsy with sufficient tumor for analysis, prior to randomization, as a minimum number needed to detect large effects among molecular biomarkers and trends for change from or association with pre-treatment and on-treatment biopsies, and clinical outcome.

As of January 2021, 96 patients had been treated but only 54 randomized. As such, it would be impossible to carry out the final analysis at 65 events. To accommodate this, we amended the trial to carry out the final analysis at 47 events (which provided 65% power under the same assumptions described above) or by January 2022 (one year after the last patient was randomized), whichever came first.

Patients receiving olaparib were monitored for toxicity according to the methods of Thall et al.^55^ starting with the first dose of olaparib with continuous monitoring planned after the 6th patient. Calculations were performed in MultcLean v2.1. Denote the probability of extreme toxicity by q_E_, where q_E_ denotes the probability of extreme toxicity (ETOX). ETOX was defined as any ≥ Grade 3 adverse event deemed possibly, probably or definitely attributable to study treatment during the first 8 weeks of olaparib except for: any Grade 3 AE alleviated or controlled by appropriate care within 14 days of onset; Grade 4 neutrophil count decrease <5 days; Grade 3 platelet count decrease without hemorrhage of any duration; Grade 3 or 4 lymphocyte count decrease of any duration; Isolated Grade 3 or 4 electrolyte imbalances/abnormalities not associated with clinical sequelae and corrected with supplementation/appropriate management; Grade 3 or 4 alkaline phosphatase increase attributable to disease response of any duration; Grade 3 or 4 asymptomatic amylase or lipase increases of any duration. We assumed q_E_ ∼ beta (0.60, 1.4). Our stopping rule was given by the following probability statement: Pr(q_E_ ∼ > 0.30 | data) >0.85. That is, we would stop the trial if, at any time during the study, we determined that there was > 85% chance that the ETOX rate was more than 30%. The stopping boundaries for this toxicity rule were to terminate the trial if the number of patients having at least one of these events compared to the number or patients who have received olaparib exceeded prespecified boundaries throughout the trial.

PFS and OS estimates from the time of randomization for the 2 randomized arms were estimated by the methods of Kaplan and Meier. The 2 randomized treatment arms were compared by a log rank test. The differences in select genomic alterations between pre-treatment and on-treatment biopsies were tested for association with PFS with Cox Proportional Hazards regression. PSA and RECIST best overall response rates will be reported separately for induction and for each randomized arm. Exploratory identification of meaningful changes among biopsies with classification and regression tree (CART) analysis were planned but not performed due to sample size constraints. Descriptive tables are provided for adverse events by grade and attribution for the cabazitaxel/carboplatin for all patients and, separately, for patients receiving olaparib maintenance vs observation. The number of cycles pre- and post-randomization are reported for each arm. A lack of statistical significance will not prevent further development of this combination if markers provide evidence of activity and a positive trend towards improvement is seen. The study was monitored by the Regulatory Personnel in the MDACC Department of Genitourinary Medical Oncology following a protocol-specific monitoring plan.

#### Histology, clinical IHC and NGS assays

Chart review of all patients that participated in this study was performed. Pathology reports were reviewed and reported histology recorded. Clinical somatic and/or germline DNA sequencing results were recorded if they had been obtained per standard clinical care. In addition, CLIA-approved immunohistochemistry results for AVPC proteins, including TP53, RB1, and PTEN were also recorded if available. These analyses were exploratory and not prespecified in the protocol.

#### NanoString analyses of tumor biopsies

Image-guided sampling of tumor deposits were mandated at baseline (within 14 days of starting chemotherapy) and at the time of randomization (after completing chemotherapy and within 14 days of starting olaparib or observation), and optional at the time of disease progression. Fresh frozen OCT compound-embedded tissue samples were cut into 4-μm thick sections with a cryostat. RNA was extracted by the classic method using Trizol reagent (Thermo Fischer Scientific, USA) according to the manufacturer’s instructions. RNA quality and quantity were assessed using the Nanodrop Spectrometer (ND-Nanodrop1000, Thermo Fisher Scientific, Wilmington, MA, USA). For the assay, 100 ng of RNA was used to detect gene expression using the nCounter Tumor Signaling 360 panel along with custom CodeSet. nCounter Digital Analyzer was used to tabulate the counts of the reporter probes and for further analysis raw data output was imported into nSolver (http://www.nanostring.com/products/nSolver). Normalization was performed using the nSolver Advanced data analysis package. Expression values were log2 transformed, following Nanostring’s Gene Expression Data Analysis Guidelines. Genes with low variance (defined as < 1e-08) were removed for each analysis. Gene set enrichment analysis (GSEA) was performed using the fgsea package in R. For the GSEA analysis, pathways with less than eight genes were removed, resulting in 47 hallmark pathways. Novel transcriptomic signatures of interest (DDR-M and arginine metabolism) were also assessed. The adjusted p-value, corrected for multiple comparisons using the Benjamini-Hochberg (BH) procedure, and the normalized enrichment score were utilized to summarize the GSEA results. Differences in the expression of individual genes were assessed using Welch’s t-test (for cross group comparisons) or paired t-test (for pre/post comparisons). Normalized NanoString data is presented in Table S6. Normalized mRNA values listed with a value of 4.3219281 were below the limit of detection. Lists of genes included on the NanoString array, as well as leading edge arginine metabolism genes, the KEGG arginine metabolism signature genes, and arginine metabolism genes derived from KEGG arginine metabolism signature that were included in the NanoString array are shown in Table S7. These analyses were exploratory and not prespecified in the protocol.

#### Animal studies

This animal study was approved by and conducted under the Institutional Animal Care and Use Committee at the University of Texas MD Anderson Cancer Center (MDACC). The development and characterization of the patient-derived xenograft (PDX) MDA PCa-144-13 (144-13) have been described.^30^ Eight-to-ten-week-old male NOD SCID mice obtained from Charles River laboratory were implanted subcutaneously in the flank region with 144-13 tumor tissue. Mice were randomized when tumors reached a palpable volume of 200 mm^3^ and treated with weekly intraperitoneal injections of vehicle (n=5) or carboplatin (20 mg/kg) + cabazitaxel (8 mg/kg) (n=10) for two weeks. Tumor growth was monitored using a digital caliper and the volume was calculated using the following formula: tumor size = (Length[mm]∗Width[mm]^2^)/2. Changes in tumor volume and survival were measured from the start of treatment and compared across the groups. Animals were euthanized when tumor volume reached 1,500 mm^3^ or body weight loss exceeded 15%. At euthanasia, fresh frozen tumor tissue was collected where possible for metabolomics. *Syngeneic experiments:* 5-week-old male C57BL/6J mice used in both TRAMP-C2 and DVL3 models were purchased from Jackson Laboratory and were quarantined for one week before the experiment. TRAMP-C2 cells were purchased from ATCC and grown in Dulbecco’s Modified Eagle Medium (DMEM) containing 5% FBS (Sigma-Aldrich), 5% Nu-Serum IV (CORNING), insulin (CORNING), and 0.01 nM dihydrotestosterone (DHT, Steraloids, Inc). DVL3 cells were provided by Dr. Ian Mills (University of Oxford) and grown in RPMI medium (Corning) supplemented with 10% FBS, 2 mM L-glutamine (Corning) and 100 nM DHT. Castrations were conducted one week before DVL3 tumor cell inoculation. TRAMP-C2 or DVL3 cells were resuspended in phosphate-buffered saline (PBS) and mixed 1:1 with Matrigel (CORNING). 1 × 10^6^ cells in 100 μl mixture was subcutaneously injected into the right flank of each mouse. Tumor volumes and body weight were monitored 2 times per week, and the tumor volumes were calculated as (width)^2^ × length/2. Mice were randomized into four groups when tumor volumes reached about 100 mm^3^. The carboplatin (20 mg/kg) and cabazitaxel (8 mg/kg) (CC, MedChemExpress, USA) treatment group of mice were intraperitoneally injected weekly for two times only. The ADI-PEG20 (Polaris Pharmaceuticals) treated group received a weekly dose of 53.3 IU/kg intramuscularly injected until the endpoint. The CC + ADI-PEG20 group received the same doses and same treatment regimens of carboplatin, cabazitaxel and ADI-PEG20. The vehicle group of mice were intraperitoneally injected with the same vehicles as for carboplatin, cabazitaxel and ADI-PEG20. Mice in the TRAMP-C2 model were euthanized at the endpoint when the total volume of the tumor reached 1500 mm^3^.

#### Metabolomics

Metabolomics analysis of MDA-PCa-144-13 tumor tissues from vehicle and carboplatin + cabazitaxel relapse groups was performed using ion chromatography–mass spectrometry (IC/MS) by the MDACC Metabolomics Core as previously described.^35^ Briefly, to determine the relative abundance of polar metabolites in mouse tissue samples, extracts were prepared and analyzed by ultra-high-resolution mass spectrometry (HRMS). Approximately 20-30 mgs of tissue were snap frozen in liquid nitrogen, then homogenized with Precellys Tissue Homogenizer. For metabolomics analysis of MDA-PCa-144-13 cells, cells were seeded at a density of 2 × 10^6^ cells per 10 cm^2^ dish and allowed to adhere for 48 h prior to treatment. Cells were then treated with carboplatin (125 μM), cisplatin (10 μM), and/or ADI-PEG20 (1 μM) for 72 h. Following treatment, cells were harvested by centrifugation, washed with cold PBS, and snap frozen in liquid nitrogen prior to metabolite extraction and analysis.

For IC/MS analysis, metabolites were extracted using ice-cold 80/20 (v/v) methanol/water with 0.1% ammonium hydroxide. Extracts were centrifuged at 17,000 g for 5 min at 4°C, and supernatants were transferred to clean tubes, followed by evaporation to dryness under nitrogen.

Dried extracts were reconstituted in deionized water, and 10 μL was injected for analysis by ion chromatography (IC)-MS. IC mobile phase A (MPA; weak) was water, and mobile phase B (MPB; strong) was water containing 100 mM KOH. A Thermo Scientific Dionex ICS-6000+ system included a Thermo IonPac AS11 column (4 μm particle size, 250 x 2 mm) with column compartment kept at 35°C. The autosampler tray was chilled to 4°C. The mobile phase flow rate was 360 μL/min, and the gradient elution program was: 0-5 min, 1% MPB; 5-25 min, 1-35% MPB; 25-39 min, 35-99% MPB; 39-49 min, 99% MPB; 49-50, 99-1% MPB. The total run time was 55 min. To assist the desolvation for better sensitivity, methanol was delivered by an external pump and combined with the eluent via a low dead volume mixing tee. Data were acquired using a Thermo Orbitrap IQ-X Tribrid Mass Spectrometer under ESI negative ionization mode.

All the raw data files were imported into Thermo Trace Finder 5.1 software for final analysis. The relative level of metabolites was normalized by tissue weight or total peak intensity. Liquid chromatography (LC)-MS-based metabolomics of MDACC PDXs of AR+ non-AVPC adenocarcinoma (MDA-PCa-133-4, MDA-PCa-170-1, MDA-PCa-183, MDA-PCa-274) and AVPC (MDA-PCa-144-13, MDA-PCa-155-2, MDA-PCa-146-10, MDA-PCa-177-0, MDA-PCa-205-6, MDA-PCa-166-1)^27^^,^^28^ as well as AR-targeted therapy-induced, isogenic models of mCRPC adenocarcinomaàNEPC transition (LT331 vs LTL331R PDX^26^ and V16D vs 42D cell models^25^ was performed by the Baylor College of Medicine (BCM) CPRIT Cancer Proteomics and Metabolomics Core. This targeted analysis identified amino acid, glycolysis, pentose phosphate pathway and TCA cycle metabolites. Samples were processed and analyzed as previously described.^56^^,^^57^

Metabolic set enrichment analysis was applied on the acquired metabolomics datasets using MetaboAnalyst 6.0 (https://www.metaboanalyst.ca/MetaboAnalyst/). This analysis used quantitative enrichment analysis method and the human KEGG database to identify significantly enriched pathways across the models and treatment groups.

#### Cell viability assays

Cisplatin, carboplatin and cabazitaxel were obtained from the MD Anderson Cancer Center Ambulatory Treatment Center. ADI-PEG20 (specific activity, 5-10 IU/mg) was obtained from Polaris Pharmacologies, Inc (San Diego, CA). The NCI-H660 and MDA-PCa-144-13 cell lines were gifted from the laboratory of Guocan Wang at MDACC and grown in RPMI-1640 (supplemented with insulin, transferrin, sodium selenite, hydrocortisone, beta-estradiol, HEPES, L-glutamine, L-glucose, sodium pyruvate and 5% FBS). Cell lines used for this study were authenticated prior to any experiments using short tandem repeat analyses via the MD Anderson Cytogenetics and Cell Authentication Core, and cells were routinely tested for Mycoplasma before cryopreservation and during experiments (every 6 weeks). Cell lines were used within 10 passages from thawing.

Cell viability was measured with a fluorometric test as previously described.^58^ Briefly, 1 × 10^4^ cells were seeded in a final volume of 0.2 mL per well in 96-well flat-bottom microtiter plates and were treated for 72 hours. We used the fluorometric resazurin reduction method (CellTiter-Blue; Promega) to evaluate the sensitivity of treated cells using drugs and doses as detailed in the results. Fluorescence (560Ex/590Em) was determined using a luminometer (BioTek Instruments, Synergy H4). The percentage of viable cells was calculated by using the linear least-squares regression of the standard curve. Fluorescence was determined for replicates (n=3) per treatment condition, and cell viability in treated cells was normalized to their respective controls.

#### mRNA expression analysis by quantitative real-time PCR

MDA-PCa-144-13 cells were seeded in 10 cm^2^ culture dishes at a density of 2 × 10^6^ cells per dish and allowed to adhere for 48 h. Cells were then treated with carboplatin (125 μM), cisplatin (10 μM), and/or ADI-PEG20 (1 μM) for 24 h. Following treatment, total RNA was isolated using Aurum Total RNA mini kit (Bio-Rad) according to the manufacturer’s instruction. RNA concentration and purity were assessed spectrophotometrically, and 1 μg of total RNA was used for cDNA synthesis using the iScript^TM^ cDNA synthesis kit (Bio-Rad). Reverse transcriptase quantitative real-time PCR (RT-qPCR) was performed using 2X Universal SYBR Green Fast qPCR Mix (ABclonal) on a real-time PCR system. Relative gene expression levels were determined using a comparative threshold cycle method.

#### Immunoblot analysis

For protein expression analysis, MDA-PCa-144-13 cells were seeded and treated under the same conditions as described in mRNA expression section, except that drug treatments were carried out for 48 h. Following treatment, cells were lysed using Pierce^TM^ RIPA buffer supplemented with protease inhibitor cocktail. Total protein concentration was determined using a Pierce^TM^ BCA protein assay kit. Equal amounts of protein were resolved on 12% SDS-PAGE gels and transferred onto PVDF membranes. Membranes were blocked with 5% bovine serum albumin (BSA) and incubated with primary antibodies of ASS1, ASL, ARG1, OTC and vinculin (loading control). After incubation with HRP-conjugated secondary antibodies, protein bands were detected using enhanced chemiluminescence (Western Lightning Plus-ECL). Band intensities were quantified using Image J software and graphical representations were generated using GraphPad Prism.

### Quantification and statistical analysis

Statistical analyses were performed using GraphPad Prism (Version 10) and R was used for transcriptomic, gene set enrichment, and metabolomics analyses. Statistical details for each experiment, including exact sample size (n), statistical tests used, and significance values are provided in the corresponding figure legends and result section.

Data represented as mean ± standard error (SE) or standard error of the mean (SEM), as indicated in the figure legends. For box-and-whisker plots, boxes represent the first and third quartiles with median indicated, as specified in the relevant figures. For comparisons between two groups, unpaired two-tailed student’s tests or Welch’s t tests were used as appropriate. For comparisons involving multiple groups, one-way or two-way ANOVA followed by post hoc multiple comparison tests were used as indicated in the figure legends. Survival outcomes were analyzed using Kaplan-Meier methods and statistical significance was assessed using the log-rank (Mantel-Cox) test. Drug interaction studies were analyzed using Bliss independence scoring, with Δ synergy scores >10 considered indicative of synergistic interactions. Synergy was determined using SynergyFinder 2.0.^59^ For NanoString analyses, differential expression and pathway enrichment analyses were performed as described in the figure legends, with significance defined as nominal *P* < 0.05 where applicable.

Unless otherwise stated, all statistical tests were two sided and *P* < 0.05 was considered statistically significant. Significance thresholds are indicated in the figure legends. Sample sizes were based on established standards in the field and/or prior experience with the respective experimental models; no formal statistical methods were used to predetermine sample size unless otherwise stated. Animals were randomized to treatment groups in *in vivo* studies as indicated in the experimental design, and no additional stratification methods were applied unless specified. No data or subjects were excluded from analyses unless explicitly noted.

## Supplemental information

Document S1. Figures S1–S4 and Tables S1–S6

Table S7. NanoString and GSEA expanded analyses

Table S8. PDX + cells metabolomics expanded data

Table S9. 144-13 PDX metabolomics expanded data

Table S10. Metabolomics 144-13 cells expanded data
