# Supplementary material for: Targeting arginine metabolism overcomes chemotherapy resistance in aggressive-variant prostate cancers
Source: iScience. 2026 Jun 2;29(6):116184. doi: 10.1016/j.isci.2026.116184 (PMC13254847; doi:10.1016/j.isci.2026.116184)
Supplement: Document S1. Figures S1–S4 and Tables S1–S6 [file mmc1.pdf]

iScience, Volume *29*

Supplemental information

Targeting arginine metabolism overcomes chemotherapy resistance in aggressive-variant prostate cancers

Elavarasan Subramani, Patrick G. Pilié, Rebecca Slack-Tidwell, Paul V. Viscuse, Xianghong Kuang, Thirukumaran Kandasamy, Dominik Awad, Jenny J. Han, Licai Huang, Christine B. Peterson, Amado J. Zurita, Sumit K. Subudhi, Paul G. Corn, Rama Soundararajan, Peter Shepherd, Badrajee Piyarathna, Vasanta Putluri, Nagireddy Putluri, Arun Sreekumar, Yuzhuo Wang, Amina Zoubeidi, Iqbal Mahmud, Sara A. Martinez, Lin Tan, Philip L. Lorenzi, Sreyashi Basu, Sonali Jindal, Padmanee Sharma, Christopher J. Logothetis, Timothy C. Thompson, Daniel E. Frigo, and Ana M. Aparicio

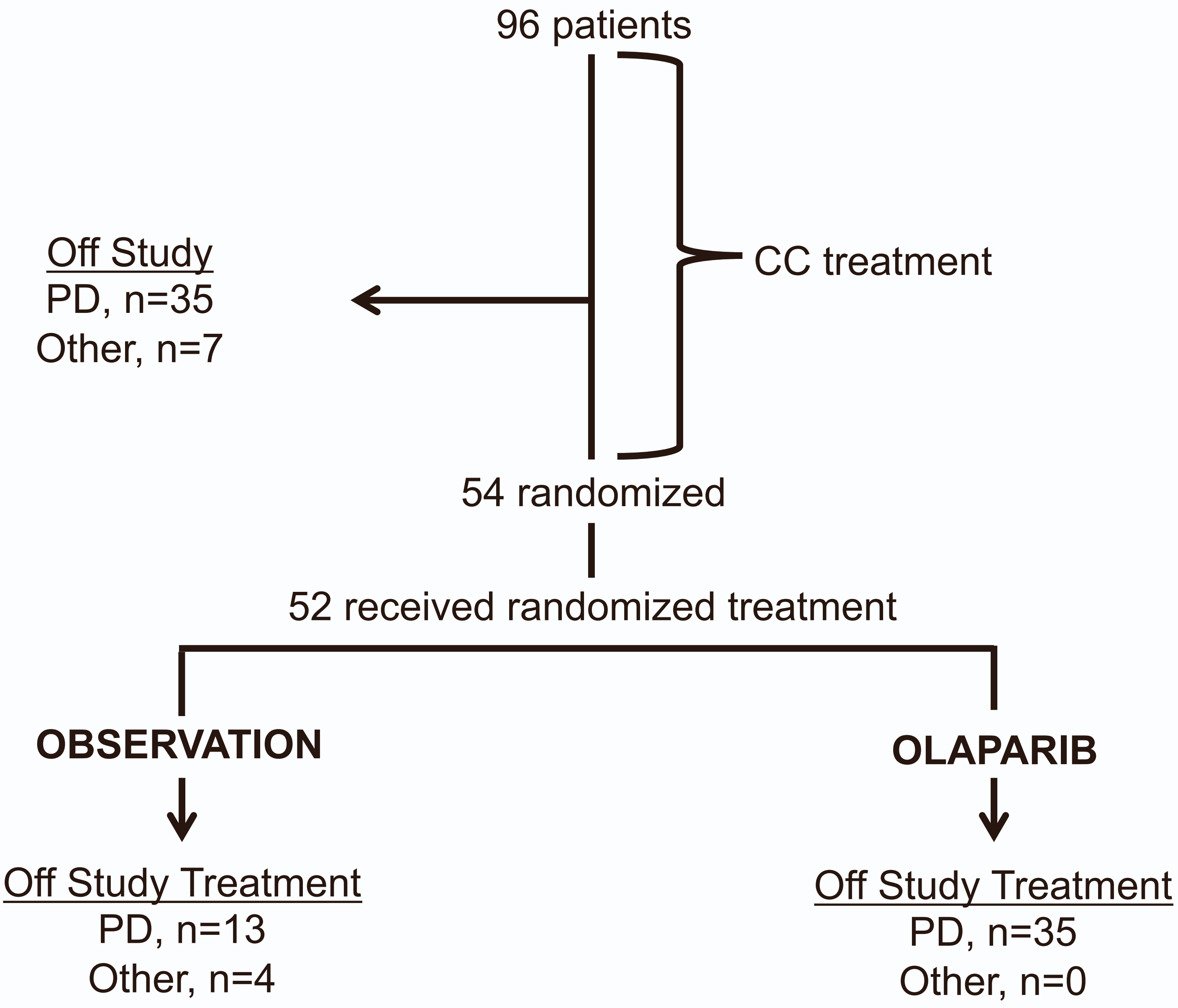

Supplementary Figure S1. Consort diagram of NCT03263650. CC, cabazitaxel plus carboplatin. PD, progressive disease.

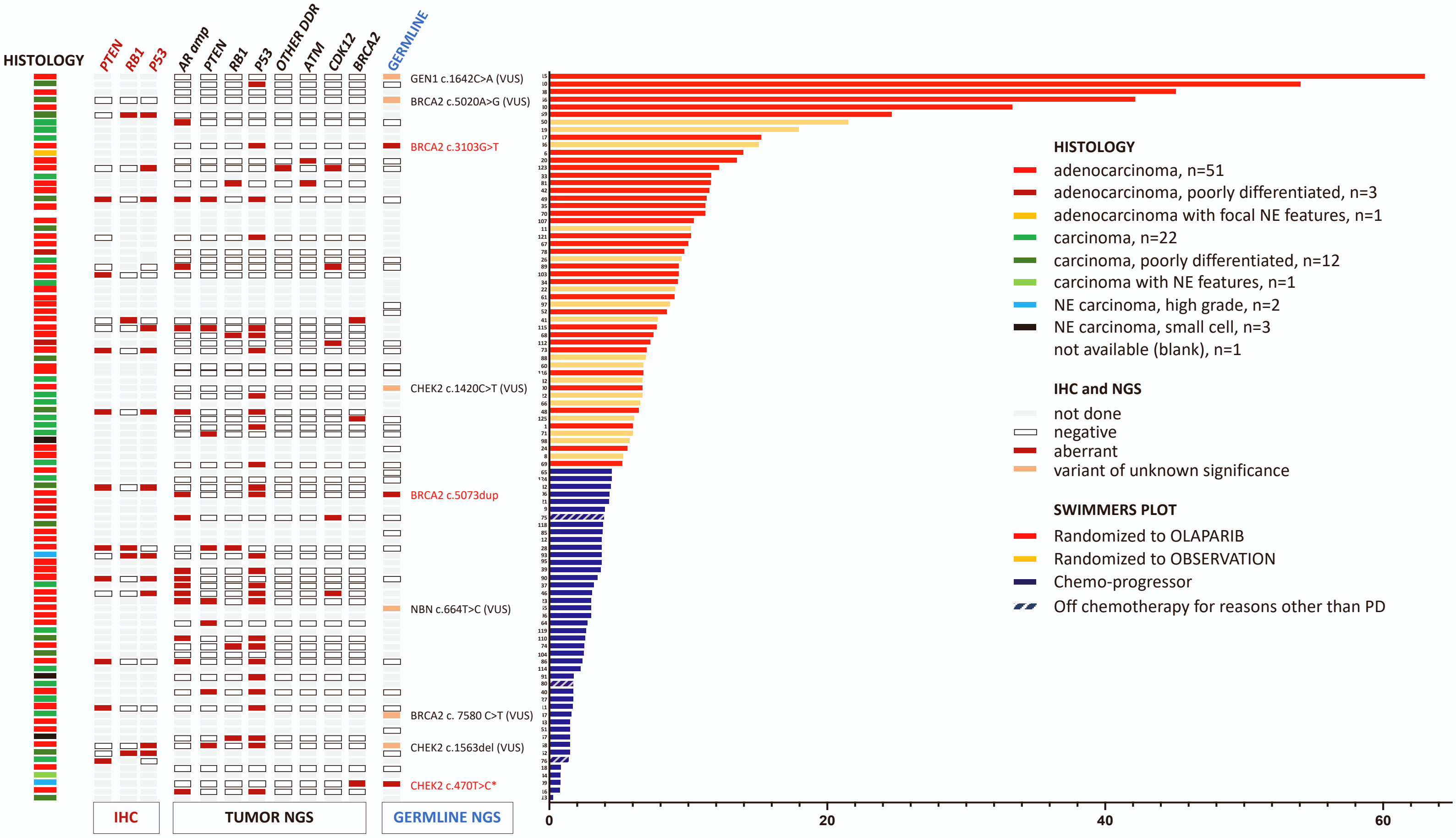

Supplementary Figure S2. PFS swimmer plot with available histology and clinical molecular somatic and germline profiling results. Presented are the swimmer plot (*right*) for patients with molecular data including histology described in pathology reports obtained within 2 years of registration, metastatic biopsy immunohistochemistry (IHC), tumor DNA next-generation sequencing (NGS) and germline NGS (*left*). *In addition to the CHEK2 c.470T>C pathogenic germline mutation, patient #109 had BRCA1 c.2597G>A (VUS); MLH3 c.4180 G>A (VUS); SMARCB1 Gain (VUS) on germline sequencing. PD, progressive disease. Of 63 patients with clinical germline and/or somatic next-generation sequencing (NGS), 12 (19%) had pathogenic variants in DDR and/or cell cycle tumor suppressor genes including *BRCA2*, *CDK12*, *ATM*, *CHEK2*, *TP53*, *RB1*, *PTEN*. Of 30 (31.2%) with both germline and somatic results available, 8 (72%) of 11 in the ChemoPD cohort and 12 (63%) of 19 in the cohort that went on to randomization had pathogenic variants in DDR genes. Nine (16.7%) of the 54 with somatic NGS results were AVPC-mDNA positive and 9 (45.0%) of 20 with clinical IHC results were AVPC-mIHC positive (combined defects in 2 or more of TP53, RB1 and/or PTEN).

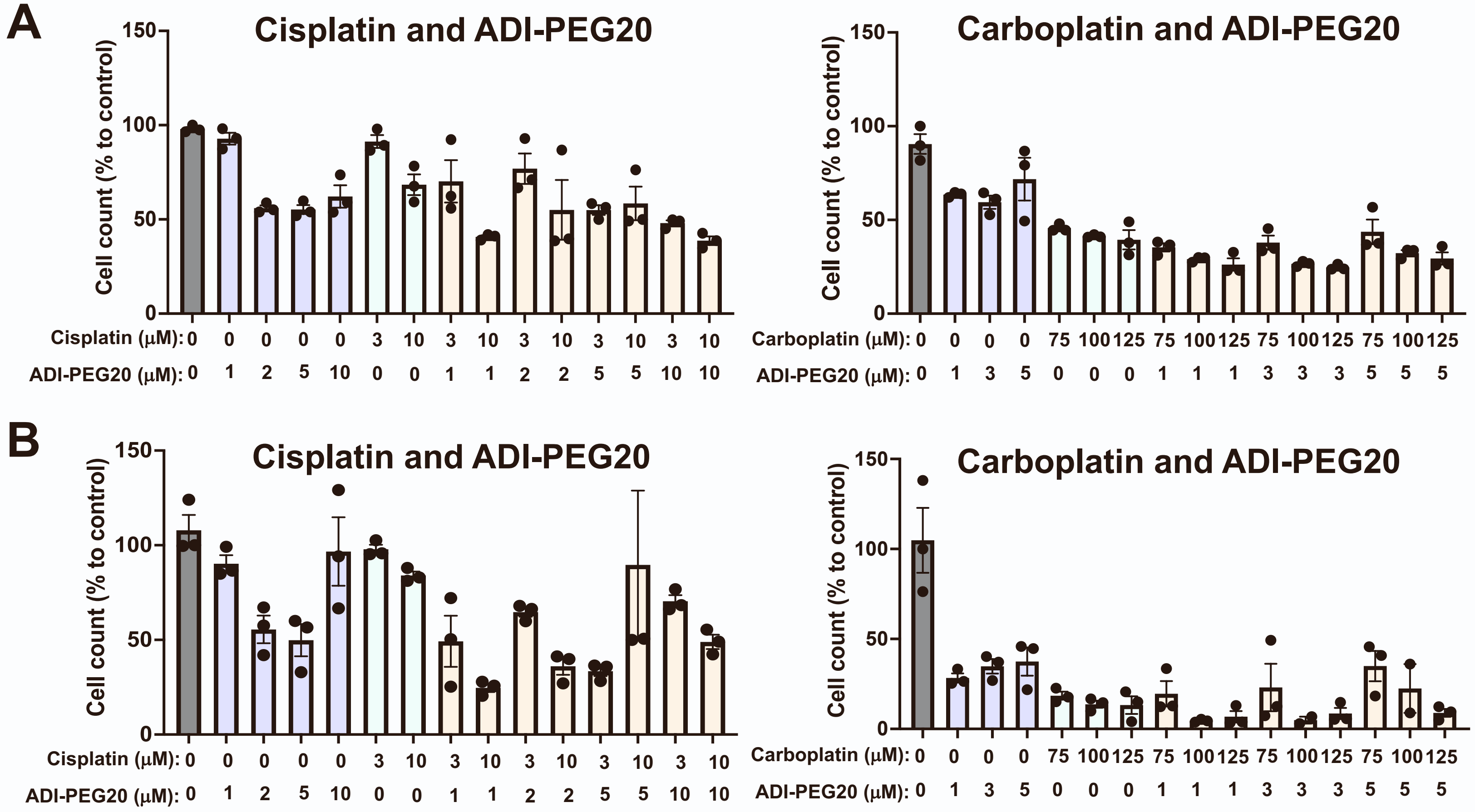

Supplementary Figure S3. Arginine depletion using pegargiminase (ADI-PEG20) sensitizes AVPC cells to platinium-based chemotherapy. (A and B) AVPC MDA-PCa-144-13 (A) or NCI-H660 (B) cell models were treated for 3 d with vehicle or increasing concentrations of cisplatin (0, 3, 10 μM) or carboplatin (0, 75, 100, 125 μM) ± increasing doses of ADI-PEG20 (0, 1, 2, 5, 10 μM). Cells were then subjected to resazurin-based cell survival assays (n=3).

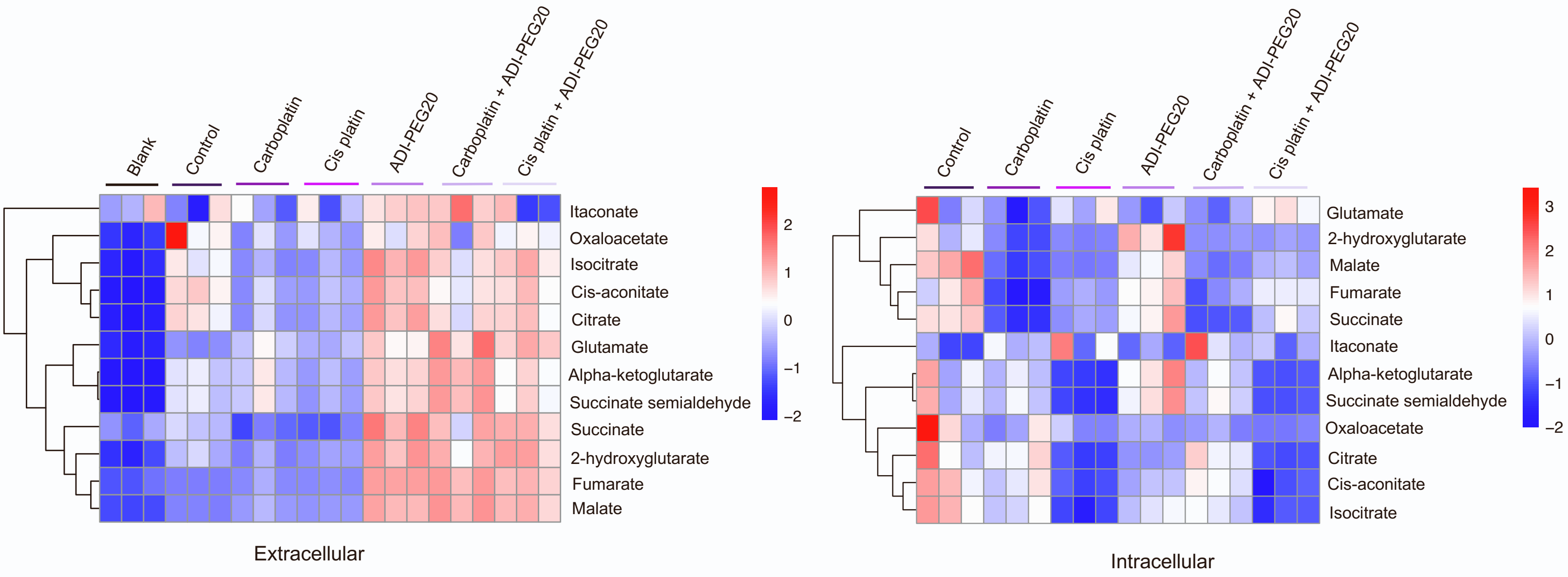

Supplementary Figure S4. Effects of arginine depletion using pegargiminase (ADI-PEG20) alone and in combination with platinum chemotherapy. MDA-PCa-144-13 cells were treated with carboplatin (125 μM), cisplatin (10 μM), and/or ADI-PEG20 (1 μM) for 72 h and subjected to IC/MS metabolomics (n=3/group). Central carbon profiles are shown here. Extracellular = media supernatent; Intracellular = cell pellet. Additional metabolomics data are shown in Figure 4 and Supplementary Table S9.

AVPC eligibility criteria for NCT03263650
1. Histologically proven small cell (neuroendocrine) prostate carcinoma
2. Exclusive visceral metastases.
3. Predominantly lytic bone metastases identified by plain x-ray or CT scan.
4. Bulky (≥ 5 cm in longest dimension) lymphadenopathy or high-grade tumor mass in prostate/pelvis.
5. Low PSA (≤ 10 ng/mL) at initial presentation (prior to androgen ablation or at symptomatic progression in the castrate-setting) plus high volume (≥ 20) bone metastases.
6. Elevated serum LDH (≥2 x ULN) or elevated serum CEA (≥2 x ULN) in the absence of other etiologies.
7. Short interval (≤ 180 days) to castrate-resistant progression following initiation of hormonal therapy.
8. Castration–resistant disease progression per RECIST in the absence of PSA values rising to ≥ 1.0ng/ml as per PCWG3 PSA progression criteria
9. Known loss or mutation (by CLIA certified molecular testing, IHC and/or DNA sequencing) in at least 2 of TP53, RB1 and PTEN defined as:<br>• IHC: Tumor samples are considered negative (and thus abnormal) for RB1 and PTEN if their labeling index is ≤ 10% and positive (and thus aberrant) for Tp53 if their labeling index is ≥ 10%, where the labeling index is defined as the percentage of positive cells, and calculated as the number of positively stained epithelial cells divided by the total number of epithelial cells, at X200 magnification.<br>• DNA sequencing: *TP53*, *RB1* and *PTEN* genes will be considered aberrant if they contain exonic nonsynonymous missense or stop-gain mutations, frameshift or non-frameshift indels (insertions or deletions), and/or copy number losses.

Supplementary Table S1. Aggressive variant prostate cancer (AVPC) eligibility criteria for NCT03263650. CEA, carcinoembryonic antigen. CLIA, Clinical Laboratory Improvement Amendments. IHC, immunohistochemistry. LDH, lactate dehydrogenase. PCWG3, Prostate Cancer Working Group 3. PSA, prostate-specific antigen. RECIST, Response Evaluation Criteria in Solid Tumors. ULN, upper limit of normal.

Patient Characteristics		All		Induction Only		Observation		Olaparib	
		N	(%)	N	(%)	N	(%)	N	(%)
**All**	**1**	**96**	**(100%)**	**42**	**(100%)**	**18**	**(100%)**	**36**	**(100%)**
Age – median (min, max)	N=96	67.0	(43.0, 86.0)	67.5	(43.0, 81.0)	66.5	(56.0, 81.0)	65.5	(47.0, 86.0)
Race/Ethnicity									
	Black/Non-Hispanic	11	(11%)	6	(14%)	2	(11%)	3	(8%)
	Other/Hispanic	4	(4%)	2	(5%)	0	(0%)	2	(6%)
	Other/Non-Hispanic	4	(4%)	1	(2%)	0	(0%)	3	(8%)
	Unknown/Not reported	2	(2%)	1	(2%)	0	(0%)	1	(3%)
	White/Hispanic	4	(4%)	3	(7%)	0	(0%)	1	(3%)
	White/Non-Hispanic	71	(74%)	29	(69%)	16	(89%)	26	(72%)
ECOG									
	0	61	(64%)	24	(57%)	9	(50%)	28	(78%)
	1/2	35	(36%)	18	(43%)	9	(50%)	8	(22%)
Prior Docetaxel									
	No	57	(59%)	18	(43%)	16	(89%)	23	(64%)
	Yes	39	(41%)	24	(57%)	2	(11%)	13	(36%)
PSA – median (IQR)	N=89	33.3	(3.4, 136.5)	37.5	(1.8, 136.5)	20.1	(1.5, 51.2)	35.6	(3.8, 176.8)
PAP – median (IQR)	N=85	7.4	(1.7, 22.0)	11.0	(2.5, 29.0)	2.3	(1.3, 4.7)	7.5	(2.2, 23.0)
CEA – median (IQR)	N=83	5.6	(2.4, 18.2)	6.2	(2.9, 14.0)	4.9	(1.7, 30.5)	3.5	(2.3, 18.2)
U NTx Mayo – median (IQR)	N=77	340.0	(155.0, 946.0)	350.0	(148.0, 1088)	310.0	(75.0, 947.0)	291.0	(155.0, 779.0)
Hgb – median (IQR)	N=93	12.1	(10.4, 13.4)	11.4	(10.3, 12.5)	12.1	(11.3, 13.4)	12.4	(10.2, 13.9)
LDH – median (IQR)	N=77	302.0	(221.0, 527.0)	381.0	(262.5, 766.5)	285.0	(245.0, 505.0)	233.5	(208.0, 367.0)
Albumin – median (IQR)	N=91	4.1	(3.9, 4.4)	4.0	(3.8, 4.3)	4.2	(3.8, 4.3)	4.2	(4.0, 4.5)
Alk Phos – median (IQR)	N=91	117.0	(82.0, 216.0)	172.0	(98.0, 287.0)	93.0	(77.0, 107.0)	102.5	(72.0, 161.0)
Alk Phos Mayo Bone – median (IQR)	N=77	21.0	(12.0, 50.0)	34.5	(14.0, 75.0)	14.5	(11.5, 26.0)	18.0	(12.0, 37.0)
Bone Metastases									
	No	20	(21%)	4	(10%)	4	(22%)	12	(33%)
	Yes	76	(79%)	38	(90%)	14	(78%)	24	(67%)

Supplementary Table S2. Baseline Characteristics by Treatment Arm on NCT03263650. Shown are baseline characteristics for all patients and broken down by assigned treatment arm.

Patient Characteristics		*ChemoPD* N (%)	*Randomized & Treated* N (%)	*P*-value
**All**		**37 (100%)**	**52 (100%)**	
Age – median (min, max)	N=89	67.0 (43.0, 75.0)	66.0 (47.0, 86.0)	0.54
Race/Ethnicity				0.74
	Black/Non-Hispanic	4 (11%)	5 (10%)	
	Other/Hispanic	2 (5%)	2 (4%)	
	Other/Non-Hispanic	0 (0%)	3 (6%)	
	Unknown/Not reported	1 (3%)	1 (2%)	
	White/Hispanic	2 (5%)	1 (2%)	
	White/Non-Hispanic	28 (76%)	40 (77%)	
ECOG				0.18
	0	20 (54%)	36 (69%)	
	1/2	17 (46%)	16 (31%)	
Prior Docetaxel				**0.004**
	No	15 (41%)	37 (71%)	
	Yes	22 (59%)	15 (29%)	
PSA – median (IQR)	N=83	32.0 (1.6, 136.5)	22.9 (3.0, 169.0)	0.79
PAP – median (IQR)	N=78	11.5 (2.5, 29.0)	5.6 (1.4, 13.5)	0.07
CEA – median (IQR)	N=76	5.7 (2.8, 10.3)	3.8 (2.3, 24.6)	0.90
U NTx Mayo – median (IQR)	N=70	340.0 (167.0, 886.0)	274.0 (125.0, 946.0)	0.66
Hgb – median (IQR)	N=86	11.4 (10.4, 12.5)	12.4 (11.3, 13.8)	**0.02**
LDH – median (IQR)	N=70	359.0 (269.0, 708.0)	260.0 (208.0, 391.0)	**0.01**
Albumin – median (IQR)	N=84	4.0 (3.7, 4.3)	4.2 (4.0, 4.4)	**0.03**
Alk Phos – median (IQR)	N=84	185.0 (97.0, 343.0)	98.0 (72.0, 153.0)	**0.003**
Alk Phos Mayo Bone – median (IQR)	N=71	48.5 (19.0, 97.0)	15.0 (12.0, 30.0)	**0.003**
Bone Metastases				**0.02**
	No	3 (8%)	15 (29%)	
	Yes	34 (92%)	37 (71%)	

Supplementary Table S3. Baseline Characteristics of "ChemoPD" vs "Randomized and Treated" patients on NCT03263650. Shown are baseline characteristics for patients that 1) progressed on chemotherapy prior to randomization (*ChemoPD*) and thus, did not continue to the ± olaparib maintenance phase and 2) made it to randomization and were treated with ± olaparib maintenance (*Randomized & Treated*).

	Induction Only (N=42)	Observation (N=18)	Olaparib (N=36)	Total (N=96)
During induction				
**PSA response**, n (%)				
PSA-CR	0 (0.0%)	1 (7.7%)	0 (0.0%)	1 (1.3%)
PSA-PR	9 (28.1%)	9 (69.2%)	21 (70.0%)	39 (52.0%)
PSA-SD	16 (50.0%)	2 (15.4%)	4 (13.3%)	22 (29.3%)
PSA-PD	7 (21.9%)	1 (7.7%)	5 (16.7%)	13 (17.3%)
Not evaluable	10	5	6	21
**RECIST response**, n (%)				
PR	1 (2.9%)	4 (23.5%)	9 (27.3%)	14 (16.7%)
SD	24 (70.6%)	13 (76.5%)	24 (72.7%)	61 (72.6%)
PD	9 (26.5%)	0 (0.0%)	0 (0.0%)	9 (10.7%)
Not available	8	1	3	12
After randomization		(N=18)	(N=36)	(N=54)
**PSA response**, n (%)				
PSA-CR		0 (0.0%)	1 (4.2%)	1 (3.1%)
PSA-PR		1 (12.5%)	1 (4.2%)	2 (6.3%)
PSA-SD		3 (37.5%)	12 (50.0%)	15 (46.9%)
PSA-PD		4 (50.0%)	10 (41.7%)	14 (43.8%)
Not evaluable		10	12	22
**RECIST response**, n (%)				
PR		5 (27.8%)	13 (38.2%)	18 (34.6%)
SD		12 (66.7%)	21 (61.8%)	33 (63.5%)
PD		1 (5.6%)	0 (0.0%)	1 (1.9%)
Not available/Not done		0	2	2

Supplementary Table S4. PSA and RECIST Responses by Treatment Arm. To be evaluable for PSA response, PSA needed to be at least 1. To have available RECIST response, patients needed target lesions and scans performed.

Most Common Grade >3 TRAEs—Induction Phase

Adverse Events - N	Grade 1/2	Grade 3/4/5	Total
**Any Event**	**48**	**44**	**92**
Anemia	40	15	55
Platelet count decreased	23	8	31
Pain	31	6	37
Fatigue	25	6	31
Diarrhea	24	6	30
Lymphocyte count decreased	17	6	23
Infection	9	6	15
Febrile neutropenia	1	6	7
Thromboembolic event	3	4	7
Hypocalcemia	10	3	13
Gastrointestinal pain	10	3	13
Hypophosphatemia	10	3	13
Dyspnea	22	2	24
White blood cell decreased	6	2	8

Most Common Grade >3 TRAEs Post—Randomization Phase

	Arm: Observation (N=18)			Arm: Olaparib (N=36)		
	Grade			Grade		
Adverse Events - N	1/2	3/4/5	Total	1/2	3/4/5	Total
**Any**	**5**	**2**	**7**	**15**	**16**	**31**
Lymphocyte count decreased	1	1	2	10	6	16
Anemia	0	0	0	4	4	8
Hyperglycemia	1	0	1	9	2	11
Fatigue	0	0	0	9	2	11
Infection	1	0	1	4	2	6

Supplementary Table S5. Most Common Grade ≥3 TRAEs. TRAE = treatment-related adverse event.

	AVPC IHC+			AVPC NGS+			DDR NGS and/or germline		
	Yes (N=9)	No (N=10)	P-value	Yes (N=7)	No (N=47)	P-value	Yes (N=12)	No (N=51)	P-value
**AVPC IHC+**									**0.03**
Yes (N=9)							0 (0%)	9 (60%)	
No (N=10)							4 (100%)	6 (40%)	
**AVPC NGS+**			>0.99						0.13
Yes (N=7)	1 (13%)	2 (20%)					0 (0%)	7 (17%)	
No (N=45)	7 (88%)	8 (80%)					12 (100%)	35 (83%)	
**Status**			0.5			0.39			0.17
Early Progressor (N=37)	5 (56%)	4 (40%)		4 (57%)	18 (40%)		3 (27%)	25 (50%)	
Randomized (N=52)	4 (44%)	6 (60%)		3 (43%)	27 (60%)		8 (73%)	25 (50%)	
**PFS from randomization**			0.86			0.58			0.96
Events/N	4/4	6/6		3/3	26/27		8/8	25/26	
Median months (95% CI)	4.3 (1.8, 18.7)	4.3 (3.0, 34.6)		2.3 (2.3, 6.2)	4.1 (2.1, 8.3)		5.2 (3.0, 9.7)	2.9 (2.1, 6.2)	
HR (95% CI) (Ref=No)	1.12 (0.29, 4.35)			1.41 (0.42, 4.76)			1.02 (0.45, 2.33)		
**PFS from induction start**			0.6			0.21			0.28
Events/N	9/9	10/10		7/7	45/47		11/12	50/51	
Median months (95% CI)	4.1 (4.0, 22.7)	7.2 (2.2, 38.8)		2.8 (2.1, 10.4)	5.9 (4.1, 8.6)		7.9 (5.6, 13.9)	4.8 (4.1, 6.2)	
HR (95% CI) (Ref=No)	1.28 (0.50, 3.33)			1.02 (0.74, 3.85)			0.69 (0.36, 1.35)		

Note, subgroup totals will not match the column or row total when there are missing data. Percentages are based on patients with available data. Percentages may not sum to 100 due to rounding.

Supplementary Table S6. DDR and AVPC associations with each other and progression.
